# Histone variants H2A.Z and H3.3 coordinately regulate PRC2-dependent H3K27me3 deposition and gene expression regulation in mES cells

**DOI:** 10.1186/s12915-018-0568-6

**Published:** 2018-09-24

**Authors:** Yan Wang, Haizhen Long, Juan Yu, Liping Dong, Michel Wassef, Baowen Zhuo, Xia Li, Jicheng Zhao, Min Wang, Cuifang Liu, Zengqi Wen, Luyuan Chang, Ping Chen, Qian-fei Wang, Xueqing Xu, Raphael Margueron, Guohong Li

**Affiliations:** 1 National Laboratory of Biomacromolecules, CAS Center for Excellence in Biomacromolecules, Institute of Biophysics, Chinese Academy of Sciences, Beijing, 100101 China; 2 University of Chinese Academy of Sciences, Beijing, 100049 China; 3 Institut Curie, PSL Research University, INSERM U934, CNRS UMR3215, 26 Rue d’Ulm, 75005 Paris, France; 4 Baoan Maternal and Child Health Hospital, Jinan University, Shenzhen, 518102 China; 5 Key Laboratory of Genomic and Precision Medicine, Beijing Institute of Genomics, Chinese Academy of Sciences, Beijing, 100101 China

## Abstract

**Background:**

The hierarchical organization of eukaryotic chromatin plays a central role in gene regulation, by controlling the extent to which the transcription machinery can access DNA. The histone variants H3.3 and H2A.Z have recently been identified as key regulatory players in this process, but the underlying molecular mechanisms by which they permit or restrict gene expression remain unclear. Here, we investigated the regulatory function of H3.3 and H2A.Z on chromatin dynamics and Polycomb-mediated gene silencing.

**Results:**

Our ChIP-seq analysis reveals that in mouse embryonic stem (mES) cells, H3K27me3 enrichment correlates strongly with H2A.Z. We further demonstrate that H2A.Z promotes PRC2 activity on H3K27 methylation through facilitating chromatin compaction both in vitro and in mES cells. In contrast, PRC2 activity is counteracted by H3.3 through impairing chromatin compaction. However, a subset of H3.3 may positively regulate PRC2-dependent H3K27 methylation via coordinating depositions of H2A.Z to developmental and signaling genes in mES cells. Using all-trans retinoic acid (tRA)-induced gene as a model, we show that the dynamic deposition of H2A.Z and H3.3 coordinately regulates the PRC2-dependent H3K27 methylation by modulating local chromatin structure at the promoter region during the process of turning genes off.

**Conclusions:**

Our study provides key insights into the mechanism of how histone variants H3.3 and H2A.Z function coordinately to finely tune the PRC2 enzymatic activity during gene silencing, through promoting or impairing chromosome compaction respectively.

**Electronic supplementary material:**

The online version of this article (10.1186/s12915-018-0568-6) contains supplementary material, which is available to authorized users.

## Background

In eukaryotic cells, chromatin organization from its basic nucleosomal structure to the more complex higher-order chromatin structures restricts the access of cellular factors/machinery to DNA. During gene transcription and other DNA-related processes, chromatin structure must be precisely regulated to allow the access of these factors/machinery to the underlying DNA template [1]. Therefore, chromatin dynamics and its epigenetic regulation are critical for the establishment and maintenance of heritable gene expression patterns during development [2]. To date, three main mechanisms, (i) DNA methylation and posttranslational modifications of histones, (ii) ATP-dependent chromatin remodeling, and (iii) the replacement of canonical histones with specific histone variants, have been identified to modulate chromatin dynamics [3]. Among them, histone variant deposition/replacement has been shown to regulate nucleosome stability and higher-order chromatin structures in a wide range of DNA-related processes, such as genome integrity, X chromosome inactivation, DNA repair, and gene transcription [4–8].

Unlike canonical histones, whose synthesis is coupled to DNA replication in S phase, histone variants are synthesized and incorporated into chromatin throughout the cell cycle. Histone variants H2A.Z and H3.3, both of which are essential for multicellular organisms [9, 10], have been demonstrated to play crucial and specific roles in regulating chromatin structure and functions during development and in diseases [11, 12]. Interestingly, H2A.Z and H3.3 were reported to play contradictory roles in nucleosome stability, gene regulation, and heterochromatin formation [12–16]. H2A.Z was linked to both transcriptional activation and repression [17]. Genome-wide studies in a variety of organisms show that H2A.Z is enriched at the promoter of inducible genes under repressed or basal expression conditions, but is subsequently removed upon transcriptional activation [18, 19]. A few recent studies further demonstrate that H2A.Z exhibits a repressive role in gene transcription [20]. In contrast, H3.3, which is deposited into transcribed genes, promoters, and gene regulatory elements, is considered as a mark of transcriptionally active genes [12, 21]. Furthermore, we previously demonstrated that H3.3 decorates enhancer regions and creates an open chromatin signature to prime genes for transcriptional activation. Additionally, H3.3-dependent recruitment of H2A.Z at the promoter regions results in chromatin compaction and poises genes for rapid transcriptional activation upon induction [22]. Of note, H3.3 has also been linked to transcriptional repression [23–25]. Indeed, H3.3 is incorporated in mES cells at the heterochromatic regions of the genome such as telomere, pericentric heterochromatin, and retroviral elements [26, 27].

Polycomb repressive complex 2 (PRC2) catalyzes the methylation (di- and tri-) of Lys27 of histone H3, whose enzymatic activity is crucial for the maintenance of gene silencing [28]. H3.3 and H2A.Z were reported to modulate PRC2 activity in mES cells [29, 30]. However, the underlying mechanisms remain largely unknown. In this study, we demonstrated that H2A.Z enhances PRC2-mediated H3K27 methylation through its effect on chromatin structure. In contrast, H3.3 counteracts the stimulatory effect of H2A.Z on chromatin compaction, which may account for its negative effect on PRC2 activity. Together, our findings uncover the important regulatory functions of histone variants H3.3 and H2A.Z in the establishment and maintenance of H3K27me3 chromatin mark at the promoter regions of developmental genes in mES cells via modulating chromatin structure.

## Results

### H2A.Z is required for the proper genome-wide distribution of H3K27me3 in mES cells

**Fig. 1:**
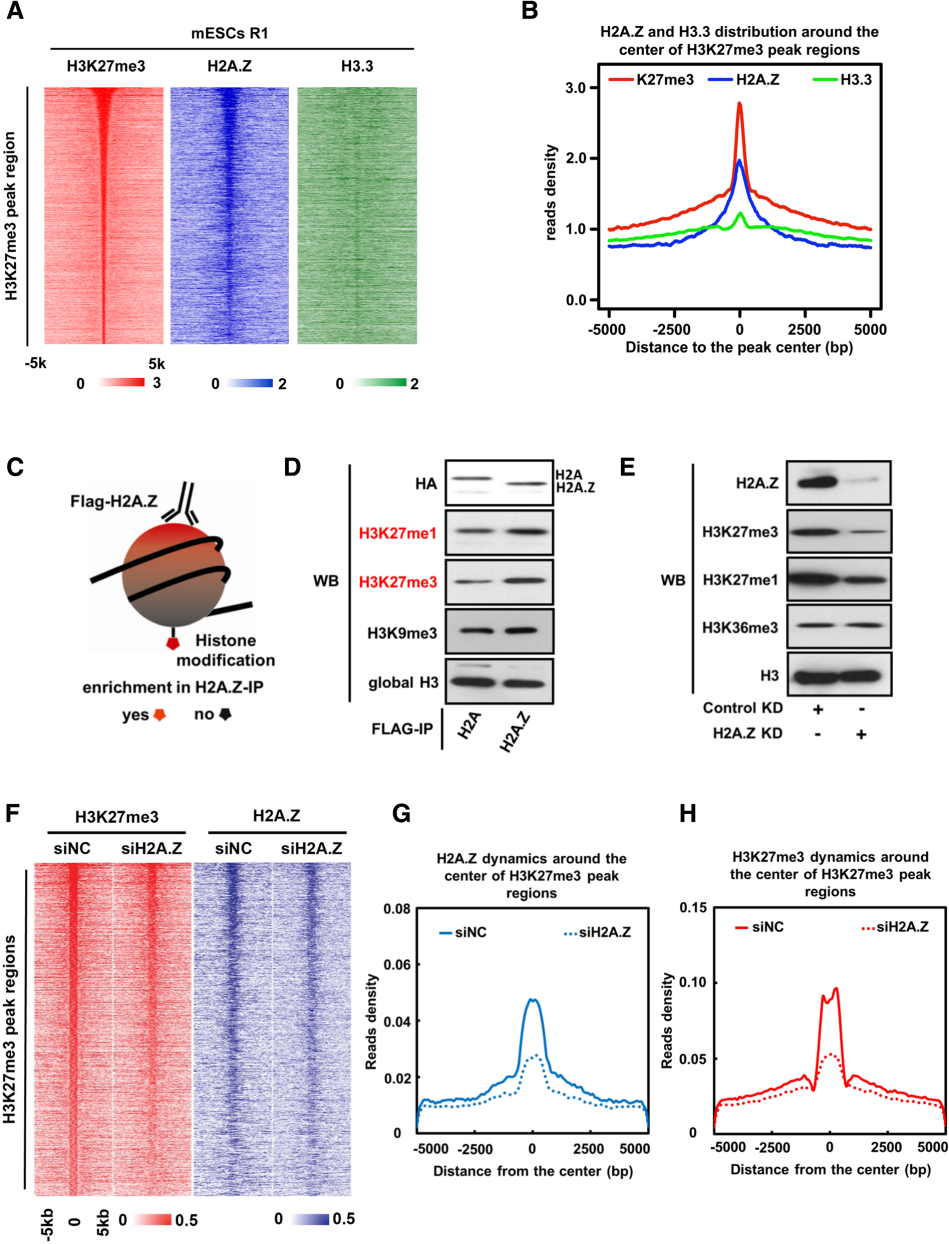
H2A.Z is required for the proper genome-wide distribution of H3K27m3 in mES Cells. **a** Heat map for distributions of H3K27me3, H2A.Z, and H3.3 at the H3K27me3-enriched regions in R1 mES cells. The *y*-axis coordinates with 4,445 H3K27me3 peaks in mES cells. The zero point of *x*-axis corresponds with the center of each H3K27me3 peak and expands from 5 kb upstream to 5 kb downstream to form H3K27me3 peak regions. **b** Scatterplot for the average reads density of H3K27me3 (red line), H2A.Z (blue line), and H3.3 (green line) at the H3K27me3-enriched regions. **c** The schematic view of mononucleosomes immuno-precipitation (Mono-IP), stable overexpressed histone H2A, or H2A.Z mouse R1 ES cell line was generated and applied here. **d** Histone modifications associated with H2A- and H2A.Z-containing mononucleosomes (marked in **c**). The histone modifications preferentially associated with H2A.Z-containing mononucleosomes are highlighted in red. **e** Western blot to analyze the dynamic changes of the global level of H3K27me3 upon knockdown of H2A.Z in mES cells. **f** Heat map for dynamic changes of H3K27me3 and H2A.Z levels at the H3K27me3-enriched regions as shown in **a** upon knockdown of H2A.Z in mES cells. **g** Scatterplot for average reads densities of H2A.Z at the H3K27me3-enriched regions (as shown in **a**) in wild-type mES cells (referred as siNC) and H2A.Z knockdown mES cells (referred as siH2A.Z). **h** Scatterplot for average reads densities of H3K27me3 at the H3K27me3-enriched regions (as shown **a**) in wild-type mES cells (referred as siNC) and H2A.Z knockdown mES cells (referred as siH2A.Z)

Recent studies have shown that histone variants H2A.Z and H3.3 may function in regulating the levels of H3K27me3 across the whole genome in mES cells [31, 32]. To investigate the underlying mechanisms, we first perform ChIP-seq analyses for H2A.Z, H3K27me3, and H3.3 in R1 mES strains. Consistent with previous results, H2A.Z showed high enrichment at H3K27me3-enriched regions (Fig. 1a, b, Additional file 1: Figure S1A, Figure S1D, and Figure S1F), suggesting that H2A.Z may have a positive role in regulating PRC2 activity as proposed previously [29, 32]. In contrast to the case of H2A.Z, only weak co-localization as well as low correlations was observed for H3.3 with H2A.Z or H3K27me3 at the regions enriched with H3K27me3 in mES cells (Fig. 1a, b, Additional file 1: Figure S1B to S1D).

To determine whether H2A.Z and H3K27me3 are present concomitantly on the same nucleosome, we analyzed the enrichment of H3K27me3 in the nucleosomes containing either H2A or H2A.Z in mES cells by mononucleosome immunoprecipitation and Western blot assays (Fig. 1c, d). Our results revealed that H2A.Z-containing nucleosomes are preferentially enriched for H3K27me3 as compared with H2A-containing nucleosomes. To investigate whether the co-localization of H2A.Z and H3K27me3 at chromatin reflects a causal link as suggested previously [29], we monitored the changes of global level of H3K27me3 in mES cells upon knockdown of H2A.Z. Of note, we failed to generate H2A.Z-knockout ES cells, suggesting that H2A.Z is essential for ES cell proliferation [33]. Knockdown of H2A.Z by RNA interference substantially reduces the global level of H3K27me3 (Fig. 1e). However, the levels of PRC2 components were unchanged in H2A.Z knocking down mES cells (Additional file 1: Figure S1G). We then analyzed the dynamic changes of H3K27me3 across the whole genome upon knockdown of H2A.Z by performing the ChIP-seq analysis. Our results showed that local H3K27me3 enrichment is also significantly reduced after knockdown of H2A.Z (Fig. 1f–h). Pearson correlation analysis showed that dynamic changes of H3K27me3 level exhibit strong positive correlations with that of H2A.Z at H3K27me3-enriched regions (*r* = 0.69). In contrast, H2A.Z deposition is not dependent on PRC2 as previously reported [34]. To extend our observation that H2A.Z positively regulates H3K27me3 to different cell context, we performed the similar ChIP-seq analyses for H2A.Z and H3K27me3 in T lymphocyte cells. The results showed that H2A.Z is highly enriched at the H3K27me3 peak regions in T cells as well (Additional file 2: Figure S2A and S2B). Furthermore, after conditional depletion of H2A.Z in CD4+ T cells, the level of H3K27me3 was significantly downregulated (Additional file 2: Figure S2C and S2D). Altogether, our results suggest that H2A.Z positively regulates PRC2 activity to maintain H3K27me3 levels through a common mechanism in both mES cells and CD4+ T cells.

### H2A.Z enhances PRC2 enzymatic activity through facilitating chromatin compaction

**Fig. 2:**
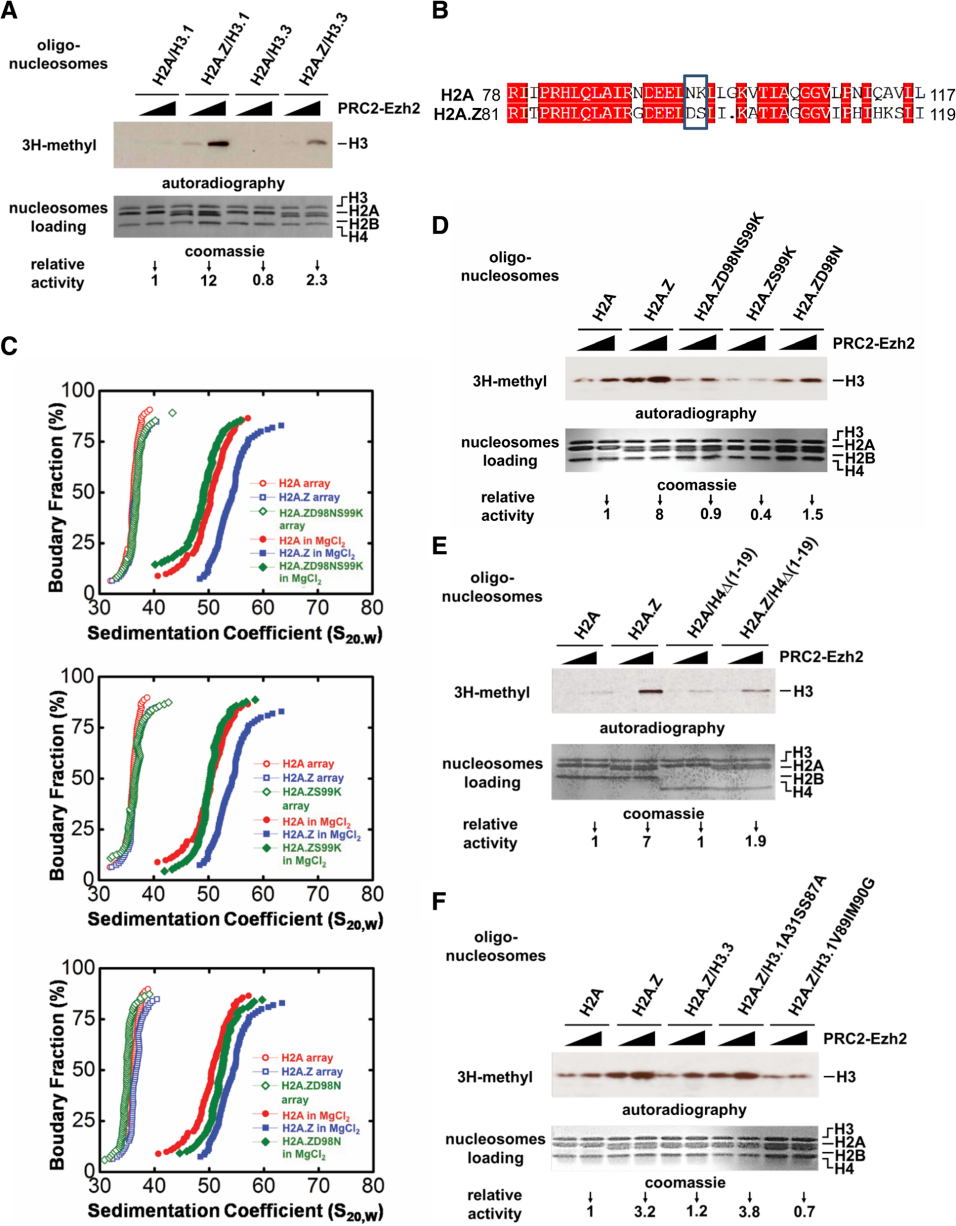
Promotion of PRC2 enzymatic activity by H2A.Z through facilitating chromatin compaction. **a** PRC2 histone methyltransferase activity in vitro (HMT assay) on the nucleosomal substrates containing a different combination of histone variants (H2A/H3.1, H2A.Z/H3.1, H2A/H3.3, and H2A.Z/H3.3). Purified PRC2 complex and isotope-labeled *S*-adenosylmethionine (3H-SAM) were applied in HMT assay. PRC2 activity was measured by auto-chromatography and a liquid scintillation counter. The relative activities of PRC2 on histone variants-containing nucleosomes were normalized with the PRC2 activity on the canonical nucleosome (H2A/H3.1-nucleosome). **b** Sequence alignment between amino acid sequences of H2A and H2A.Z, divergent residues in extended acid patch domain are highlighted with a black box. **c** Analytical ultracentrifuge assay (AUC) to monitor the effect of mutations at the extended acid patch of H2A.Z (S99K, D98N, and D98N/S99K) on the H2A.Z-enhanced chromatin compaction. **d** HMT assays to monitor the effect of mutations at the extended acid patch (S99K, D98N, and D98N/S99K) on the H2A.Z-enhanced PRC2 activities. **e** HMT assay to monitor the effect of deletion of histone H4 N-terminal domain (NTD) on H2A.Z-enhanced PRC2 enzymatic activity. **f** HMT assay to monitor the effect of mutations on H3.1 (V89M/M90G and A31S/S87A) on H2A.Z-enhanced PRC2 enzymatic activity

To investigate the mechanism underlying how H2A.Z and H3.3 coordinately modulate PRC2 activity, we monitored its histone methyltransferase activity in vitro on the four different nucleosomal substrates (H2A/H3.1, H2A.Z/H3.1, H2A/H3.3, and H2A.Z/H3.3). Interestingly, compared with the canonical histone H2A-containing oligo-nucleosomes, PRC2-mediated histone H3 methylation is substantially enhanced on oligo-nucleosomes containing H2A.Z (Fig. 2a). This H2A.Z-dependent effect is specific to PRC2 since the H3K9 methylation enzyme Suv39h1 does not show this effect (Additional file 3: Figure S3A). Importantly, H2A.Z-mediated stimulation of PRC2 activity is specific for oligo-nucleosomal substrates as no significant stimulation was observed when histone octamers or mononucleosomes were used as substrates (Additional file 3: Figure S3B and S3C). Previously, oligo-nucleosomes have been shown to be better substrates for PRC2 as compared with histone octamers and mononucleosomes [35, 36], suggesting that a specific chromatin conformation adopted by oligo-nucleosomes may be sensed by PRC2. We have shown that the incorporation of H2A.Z can facilitate nucleosomal arrays folding to a unique “ladder-like” structure in the presence of 1 mM MgCl_2_ [22]. Therefore, it is tempting to speculate that H2A.Z may regulate PRC2 activity through modulating the structure of chromatin. The extended acidic patch of H2A.Z, especially the residues Asn98 (vs. Asp in H2A) and Lys 99 (vs. Ser in H2A), has been shown to be required for higher-order chromatin folding [16, 37] (Fig. 2b). Using analytical ultracentrifugation (AUC), we confirmed that either single mutation (S99K or D98N) or double mutations (D98N/S99K) of these two key residues efficiently impairs H2A.Z-mediated chromatin compaction (Fig. 2c). We then analyzed the PRC2 activities on the corresponding substrates. Interestingly, our in vitro histone methyltransferase (HMT) assays indeed showed that either single mutation (S99K or D98N) or double mutations (D98N/S99K) of these two key residues significantly abrogate the H2A.Z-mediated stimulation of PRC2 activity (Fig. 2d). In summary, our results indicate that the extended acidic patch of H2A.Z plays a critical function in facilitating chromatin compaction, which then may positively regulate PRC2-mediated H3K27 methylation.

To further confirm the above result, we monitored the effect of deleting the histone H4 N-terminal domain (NTD), which was reported to greatly impair chromatin compaction [38], on the H2A.Z-enhanced PRC2 activity. Interestingly, the deletion of H4 NTD results in great impairment of H2A.Z-enhanced PRC2 activity (Fig. 2e). Altogether, our results suggest that H2A.Z can promote PRC2 enzymatic activity through facilitating chromatin folding to generate a preferred substrate for PRC2.

### H3.3 negatively regulates PRC2 activity by counteracting H2A.Z-mediated chromatin compaction

We have previously shown that H3.3 can greatly impair the chromatin fiber folding and counteract the H2A.Z-mediated chromatin compaction [22]. Therefore, we hypothesize that H3.3 may play a negative role in regulating the PRC2 activity. Our in vitro histone methyltransferase activity assays revealed that, compared with oligo-nucleosomes containing H2A.Z and H3.1, the replacement of H3.1 by H3.3 indeed impairs the H2A.Z-enhanced PRC2 activity (Fig. 2a). H3.3 is one of the most conserved variants in eukaryotes and has only four to five amino acid residues difference from canonical H3, including residue 31 (Ser vs. Ala) in the N-terminal tail and residues 87 (Ala vs. Ser), 89 (Ile vs. Val), and 90 (Gly vs. Met) near the beginning of the α2 helix of histone H3 [39]. Previously, we have shown that the Ile89 and Gly90 residues on H3.3 are responsible for counteracting the effect of H2A.Z on chromatin folding and that histone H3.1 containing double mutant V89IM90G, but not A31SS87A, can significantly counteract the effect of H2A.Z on chromatin folding. Consistently, as shown in Fig. 2f, our results confirmed that introducing the double mutation V89IM90G in H3.1 is sufficient to prevent H2A.Z from promoting PRC2 activity, while another double mutant A31SS87A has no consequences in this assay (Fig. 2f). Taken together, our results demonstrate that the modulation of chromatin compaction by histone variants H2A.Z and/or H3.3 may have corresponding influences on PRC2 enzymatic activity in vitro.

### H2A.Z-dependent alteration of chromatin structure and H3K27me3 deposition in mES cells

**Fig. 3:**
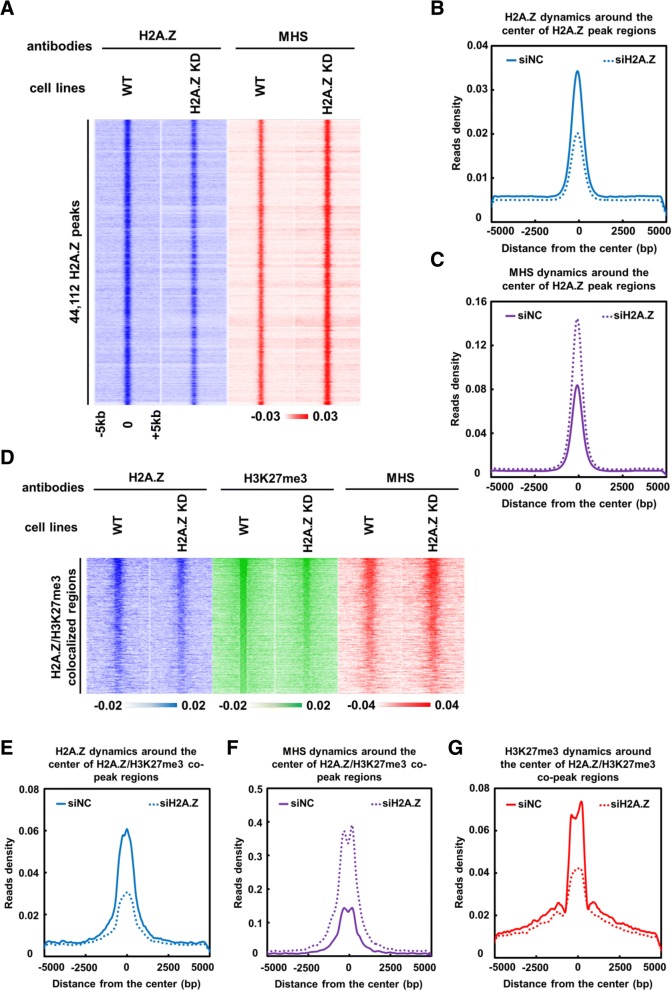
Dynamic changes of chromatin compaction and H3K27me3 deposition upon knockdown of H2A.Z in mES cells. **a** Heat map for dynamic changes of MNase hypersensitivities (MHS) and H2A.Z levels around the H2A.Z peak regions after H2A.Z knocking down in mES cells. The *y*-axis coordinates with 44,112 high confident H2A.Z peaks in mES cells. The zero point of *x*-axis corresponds with the center of each H2A.Z peak and expands from 5 kb upstream to 5 kb downstream to form H2A.Z peak regions. Chromatin accessibility was measured by MNase hypersensitive sites which could be digested by micrococcal nuclease (MNase). **b** Scatterplot for average reads densities of H2A.Z around the H2AZ peak regions (as shown in **a**) in wild-type mES cells (referred as siNC) and H2A.Z knockdown mES cells (referred as siH2A.Z). **c** Scatterplot for average reads densities of MNase hypersensitivities (MHS) around H2AZ peak regions (as shown in **a**) in wild-type mES cells (referred as siNC) and H2A.Z knockdown mES cells (referred as siH2A.Z). **d** Heat map for the dynamic changes of H2A.Z, MNase hypersensitivities (MHS), and H3K27me3 levels around H2A.Z/H3K27me3-colocalized regions after H2A.Z knocking down in mES cells. The *y*-axis coordinates with 1,400 H2A.Z/H3K27me3-colocalized peaks in mES cells. The zero point of *x*-axis corresponds with the center of each peak and expands from 5 kb upstream to 5 kb downstream to form H2A.Z peak regions. **e**–**g** Scatterplots for average reads densities of H2A.Z (panel **e**), MNase hypersensitivities (panel **f**), and H3K27me3 (panel **g**) around H3K27me3 peak regions (as shown in **d**) in wild-type mES cells (referred as siNC) and H2A.Z knockdown mES cells (referred as siH2A.Z)

To further confirm our in vitro observations in cells, we investigated the regulation of H2A.Z on chromatin structure and H3K27me3 deposition in mES cells. To this end, we adapted the protocol of MNase-seq analysis, a method used to evaluate chromatin accessibility genome-wide based on partial MNase digestion [22, 40, 41]. Additionally, as a control, genome-wide nucleosome occupancy was determined by using extensive MNase digestion assay [42, 43]. Through this method, 43,340 MNase hypersensitive sites (MHS) were obtained in mES cells. We repeated this assay upon knockdown of H2A.Z. Strikingly, the number of MHS increased to 75,477 with about 25,775 common sites in both cells (Additional file 4: Figure S4B). Even among the 25,775 common MHS, we found that chromatin structure also becomes more accessible upon knockdown of H2A.Z (Additional file 4: Figure S4C). This result indicates that loss of H2A.Z leads to a genome-wide increase of chromatin accessibility. To test the direct correlation between the dynamics of H2A.Z and chromatin structure, the dynamic change of chromatin accessibility around H2A.Z peaks was analyzed upon knockdown of H2A.Z. Interestingly, the chromatin structure around H2A.Z peaks indeed becomes more open (more sensitive to MNase-digestion) upon knockdown of H2A.Z in mES cells (Fig. 3a). These results reveal that consistent with our in vitro observations, H2A.Z is an important regulator of chromatin structure in mES cells. In addition, we found that the alteration of chromatin structure around H2A.Z peaks is strongly correlated (*r* = 0.91) with the dynamic changes of H2A.Z at genome wide in mES cells (Fig. 3b, c). Of note, our above results demonstrate that H2A.Z positively regulates PRC2 activity to maintain genome-wide H3K27me3 level (Fig. 1f–h). Next, we further analyze the dynamic changes of H2A.Z depositions, chromatin structure (MNase hypersensitivities), and H3K27me3 levels at the H2A.Z/H3K27me3 overlapping regions upon knockdown of H2A.Z. Our results reveal that the levels of H2A.Z and H3K27me3 are coordinately decreased, while MNase hypersensitivities are concordantly increased at these regions after knockdown of H2A.Z (Fig. 3d–g) and at the promoter regions of representative genes as well (Additional file 4: Figure S4D and S4E). Collectively, our biochemical and genomic analyses reveal that H2A.Z promotes PRC2 activity to maintain genome-wide H3K27me3 level through modulating chromatin structure both in vitro and in mES cells.

**Fig. 4:**
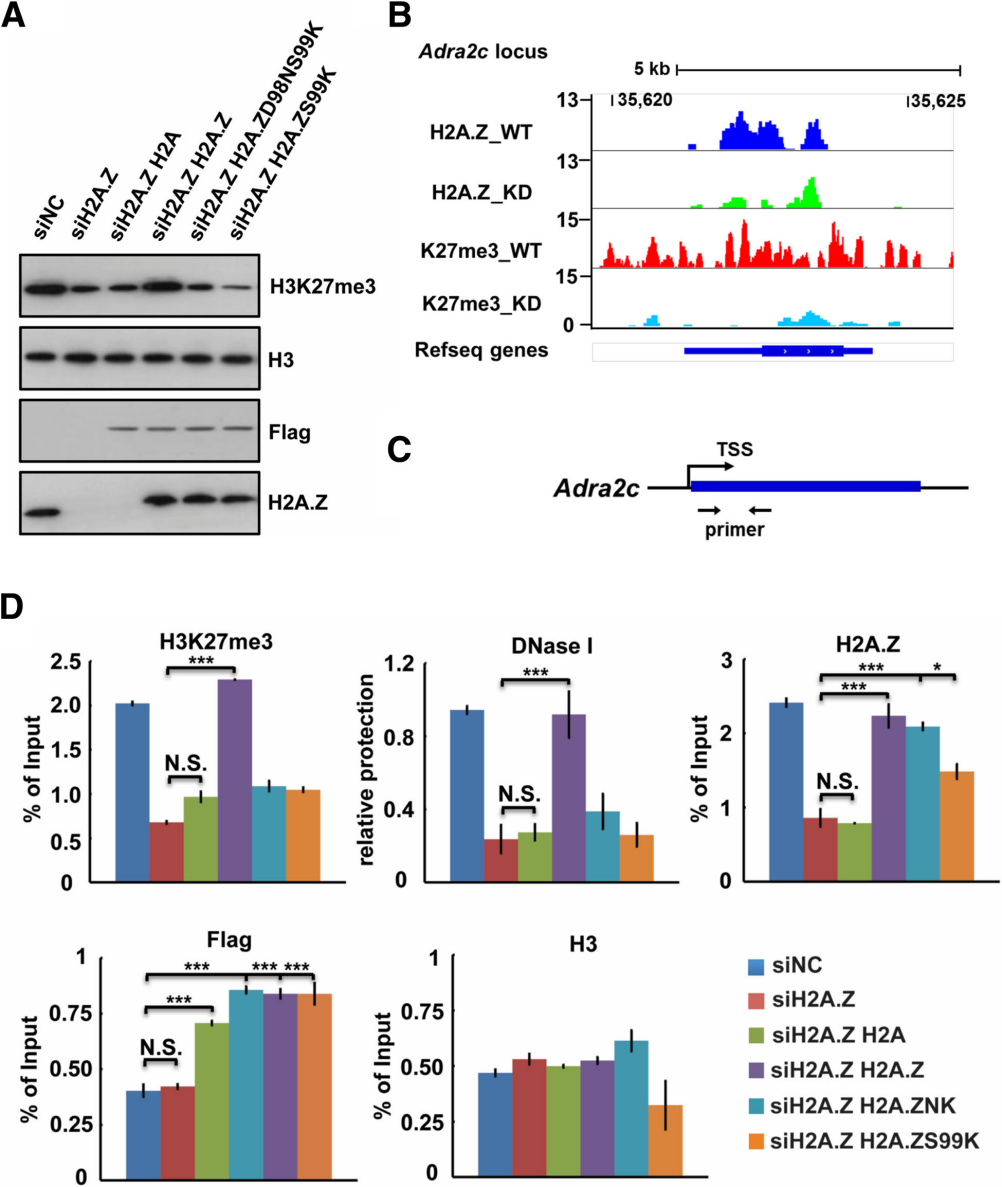
The extended acid patch of H2A.Z is important for the proper H3K27me3 level in mES cells. **a** Rescuing the global level of H3K27me3 in H2A.2 knockdown mES cells by various histones and its mutants (H2A, H2A.Z, H2A.Z D98N/S99K, and H2A.Z S99 K). **b** The dynamic changes of levels of H3K27me3 and H2A.Z in specific gene loci (*Adra2c*) upon knockdown of H2A.Z in mES cells. **c** Schematic view of the *Adra2c* promoter region. Arrows indicate the positions for primers. **d** Rescuing the deposition of H3K27me3 and the local chromatin compaction at the promoter of *Adra2c* in H2A.Z knockdown mES cells by various histones and its mutants (H2A, H2A.Z, H2A.Z D98N/S99K, and H2A.Z S99K). Levels of H3K27me3, H2A.Z, exogenous Flag-H2A.Z, and H3 at the promoter of *Adra2c* were monitored by ChIP-qPCR. The local chromatin compaction/dynamics at the promoter of *Adra2c* was analyzed by DNase protection assay via EpiQ™ kit. The *p* values were calculated with Student’s *t* test (*** < 0.01; * < 0.05; *n* = 3)

Our above biochemical results demonstrate that two key residues Asn98 and Lys99 are critical for the H2A.Z-enhanced PRC2 activity and chromatin compaction in vitro. To further confirm this result in mES cells, we performed rescue experiments with either wild-type H2A.Z or the aforementioned H2A.Z mutants (H2A.ZS99K and H2A.ZD98NS99K) in R1 mES cells. We first monitored the knockdown level of H2A.Z and insured that all H2A.Z-related rescues were expressed at a similar level (Fig. 4a). Then, we quantified the level of H3K27me3 by Western blot in the different conditions. As expected, the overexpression of wild-type H2A.Z could rescue the decreased H3K27me3 consequent to H2A.Z knockdown. In contrast, the two H2A.Z mutants deficient for chromatin compaction were unable to do so, and H3K27me3 remains at a level similar to the knockdown control (Additional file 5: Figure S5A). Since we failed to generate H2A.Z-knockout mES cells, we needed to ascertain that the results we observed were caused by the direct effect of H2A.Z knockdown and not by potential off-target effect. We therefore performed ChIP-qPCR and DNA nuclease protection (EpiQ™ kit) assays to monitor the rescues of H2A.Z, H3K27me3 deposition, and chromatin accessibility at three representative genes following knockdown of H2A.Z in mES cells (Fig. 4b, Additional file 5: Figure S5B and S5E). Our results found that both H3K27me3 and chromatin accessibility at the promoter regions of these representative genes can indeed be rescued by exogenous expression of wild-type H2A.Z (Fig. 4c, d; Additional file 5: Figure S5C and S5D; Additional file 5: Figure S5F and S5G). However, the rescue by overexpression of either H2A or H2A.Z mutants is ineffectual. To test whether the mutations of the two key residues (D98 and S99) can affect the deposition of H2A.Z at the selected target sites, we performed ChIP assays to monitor the enrichment of exogenously expressed Flag-tagged H2A.Z and its mutants at the promoter regions using antibodies specific for Flag tag and H2A.Z, respectively. Our results showed that all three forms of H2A.Z were deposited similarly at the promoter regions of representative target genes (Fig. 4d; Additional file 5: Figure S5D and S5G), indicating that the mutations of these two key residues (D98 and S99) do not affect the deposition of H2A.Z. Altogether, our results further confirm that H2A.Z promotes PRC2 activity to maintain H3K27me3 level likely through modulating chromatin structure in mES cells.

### Coordination between H3.3 and H2A.Z in regulating H3K27me3 deposition in mES cells

**Fig. 5:**
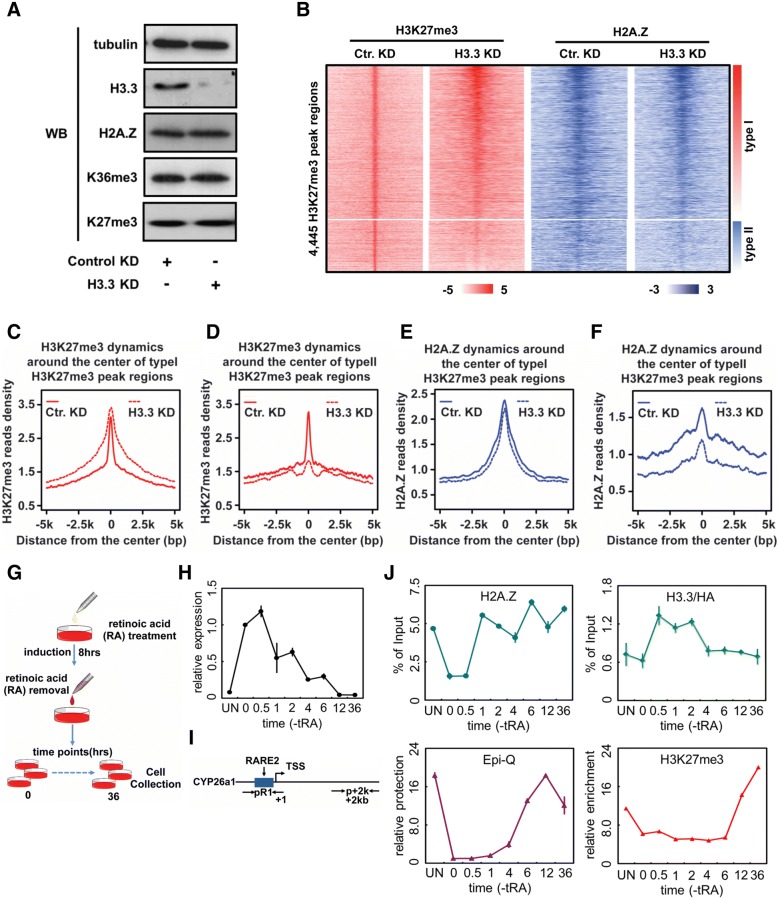
Coordination between H3.3 and H2A.Z in regulating H3K27me3 deposition in mES cells. **a** Western blot to analyze the dynamic changes of H2A.Z and H3K27me3 protein level upon knockdown of H3.3 in mES cells. No change was observed on the global levels of H2A.Z or H3K27me3 after H3.3 knocking down in mES cells. **b** Heat map for the dynamic changes of H3K27me3 and H2A.Z levels upon H3.3 knocking down in mES cells. Cluster analysis was performed for the dynamic changes of both H3K27me3 and H2A.Z levels upon H3.3 knocking down in mES cells. Two different subgroups were highlighted by a red box (type I, top part) and blue box (type II, bottom part). The *y*-axis coordinates with 4,445 H3K27me3 peaks in mES cells. The zero point of *x*-axis corresponds with the center of each H3K27me3 peak and expands from 5 kb upstream to 5 kb downstream to form H3K27me3 peak regions. **c**–**d** Scatterplots for average reads densities of H3K27me3 at type I (panel **c**) and type II (panel **d**) H3K27me3 peak regions (as shown in **b**) in wild-type mES cells (Ctr. KD) and H3.3 knockdown mES cells (H3.3 KD). **e**–**f** Scatterplots for average reads densities of H2A.Z at type I (panel **e**) and type II (panel **f**) H3K27me3 peak regions (as shown in **b**) in wild-type mES cells (Ctr. KD) and H3.3 knockdown mES cells (H3.3 KD). **g** Schematic procedure of all-trans retinoic acid (tRA) cessation experiment, the transcription of CYP26a1 was agitated by adding tRA and then was gradually ceased after tRA removal. **h** RT-qPCR analysis of the cessation of CYP26a1 expression during tRA withdrawal. **i** Schematic view of the CYP26a1 promoter region (+ 1) and gene body region (+ 2 kb). The positions for primers are indicated by arrows. **j** Dynamics of H2A.Z (upper left), H3.3 (upper right, represented by HA), chromatin structure/compaction (bottom left), and H3K27me3 (bottom right) at the promoter region of CYP26a1 during tRA cessation

H3.3 is generally considered as a marker of active transcription [12]. However, several recent studies reported that H3.3 might also play important roles in heterochromatin and PRC2-mediated gene silencing [23, 30]. Our in vitro biochemical data showed that H3.3 may negatively regulate PRC2-dependent H3K27 methylation via impairing chromatin compaction. To address the possible functions of H3.3 in PRC2-mediated H3K27 methylation and gene silencing in mES cells, we first analyzed the changes of global level of H3K27me3 and H2A.Z upon knockdown of both H3.3B and H3.3A. Both H3K27me3 and H2A.Z levels remain unchanged after knockdown of H3.3 in mES cells as shown by Western blot (Fig. 5a). Next, employing ChIP-seq analysis, we measured the dynamic changes of the enrichments of both H3K27me3 and H2A.Z at genome wide after depletion of H3.3. In general, H3K27me3 displays bi-directional dynamic changes in response to H3.3 knocking down. In the majority of H3K27me3 peak regions, the H3K27me3 level is obviously upregulated after the depletion of H3.3 (Fig. 5b, c), which largely agrees with our in vitro biochemical results that H3.3 negatively regulates PRC2 activity (Fig. 2a). We refer to these peaks as type I region. In contrast, for a subset of H3K27me3 peak regions, we observed a significant decrease of the H3K27me3 level upon loss of H3.3 (Fig. 5b, d). This subset of peaks is referred to as type II region. We then compared the dynamic changes of H2A.Z levels between these two regions upon loss of H3.3. Unlike the type I regions in which H2A.Z enrichment remains almost unchanged, in the type II set of genomic regions, the loss of H3.3 leads to an obvious decrease of H2A.Z (Fig. 5b, e, f). The simultaneous downregulations of H2A.Z and H3K27me3 in the type II regions indicate that H3.3 and H2A.Z may coordinately regulate H3K27me3 deposition in mES cell. Of note, we previously have shown that H3.3 at enhancer region coordinately regulates the distal deposition of H2A.Z at the promoter region to regulate chromatin structure and gene transcription [22]. Therefore, we hypothesize that H3.3 may coordinately regulate the deposition of H2A.Z at the type II regions, which subsequently facilitates chromatin compaction and promotes the PRC2 activity for H3K27me3.

To further decipher the biological function of H3.3 in PRC2-mediated H3K27 methylation and gene silencing, we next perform gene annotations of these type I and type II genomic regions. Interestingly, genes highly enriched at type I regions are involved in the development process (Additional file 6: Figure S6A), indicating that H3.3 might regulate developmental genes through coordinating with H2A.Z to impact PRC2-dependent H3K27me3 deposition. Previously, we found that H3.3 and H2A.Z coordinately regulate the expression of all-trans retinoic acid (tRA)-regulated genes in mES cells [22]. Importantly, several tRA-regulated genes are also found in the above subgroup of genes (type I). We therefore investigate how H2A.Z and H3.3 deposition, H3K27me3 enrichment, and gene transcription are temporally orchestrated in CYP26a1, one of the tRA-regulated genes. CYP26a1 is enriched for H3K27me3 in undifferentiated mES cells, and its expression is induced upon tRA treatment before going back to its initial level after removal of tRA. To mimic the establishment of gene repression mediated by PRC2, we investigate the inactivation of CYP26a1 gene that has been fully activated by tRA (8 h) and then by the withdrawal of tRA (Fig. 5g). Our reverse transcription quantitative PCR (RT-qPCR) result showed that transcription of CYP26a1 is gradually decreased after the removal of tRA (Fig. 5h). The dynamics of H2A.Z, H3.3, H3K27me3, and chromatin accessibility at the promoter and gene body of CYP26a1 were monitored by ChIP-qPCR and DNA nuclease protection assays, respectively (Fig. 5i, j and Additional file 6: Figure S6B). At the chromatin level, two phases were observed in the promoter region during the inactivation of CYP26a1 after the withdrawal of tRA (Fig. 5i, j). During the first phase (0–2 h), H2A.Z and H3.3 are rapidly re-deposited, while no change of H3K27me3 enrichment and chromatin accessibility is observed (Fig. 5j). During the second phase (2–36 h), H2A.Z remains constant and H3.3 goes back to its original level, while chromatin accessibility is reduced and, subsequently, H3K27me3 enrichment is restored (Fig. 5j). In contrast, no such dynamic patterns were observed at the gene body of CYP26a1 for either H2A.Z, H3.3, H3K27me3, or chromatin accessibility during this process (Additional file 6: Figure S6B). Therefore, our results from time course ChIP and DNA nuclease protection assays suggest that the local chromatin structure at the promoter region of Cyp26A1 is highly correlated with the dynamics of H2A.Z and H3.3 (Fig. 5j). Taken together, our results support the coordination of histone variants H3.3 and H2A.Z in regulating the deposition of H3K27me3 at the promoters of PRC2-targeted genes through the modulation of chromatin structure in mES cells.

## Discussion

**Fig. 6:**
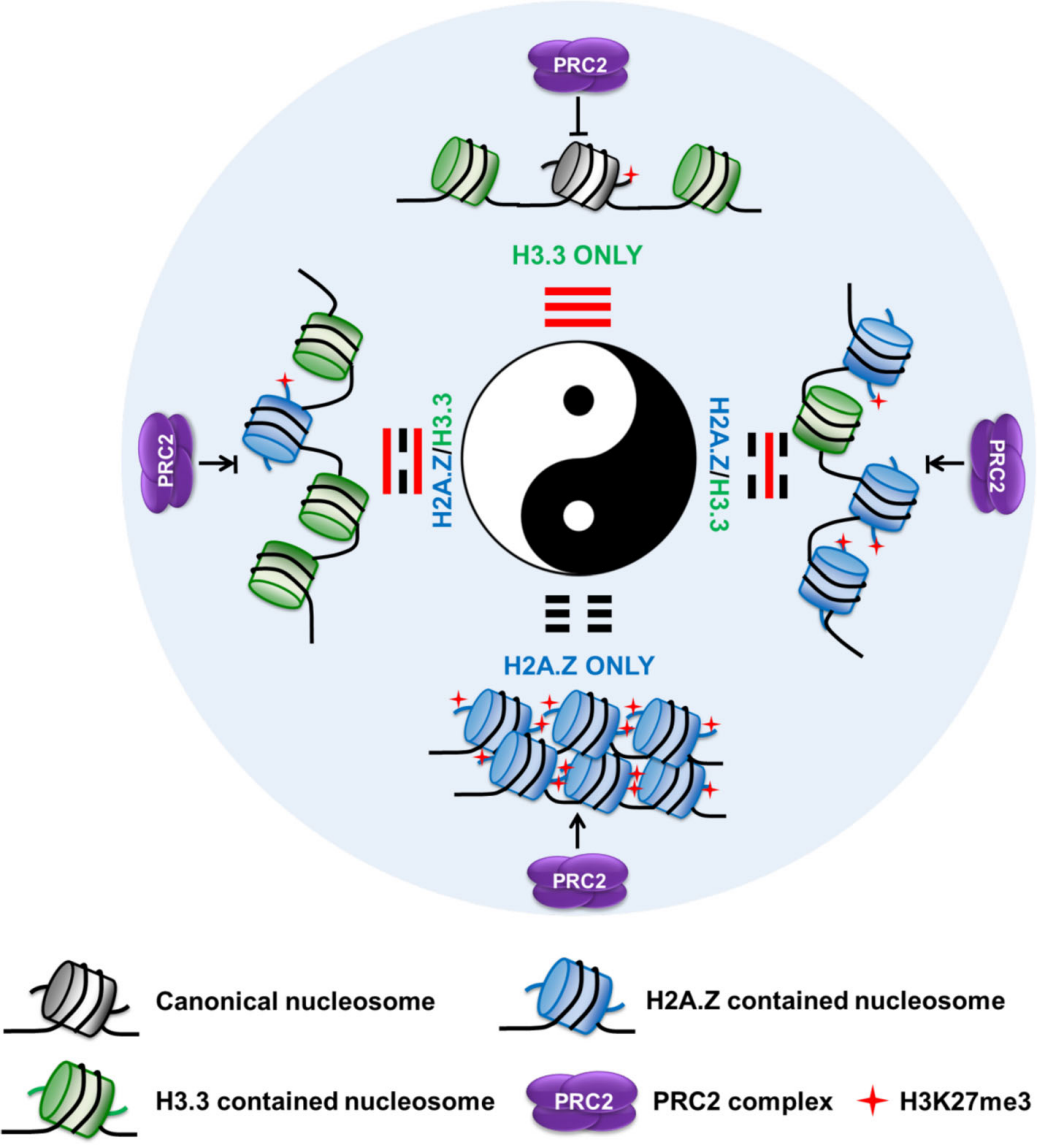
Model for the balance between H2A.Z and H3.3 in regulating H3K27me3 deposition by PRC2 in mES cells. In this model, H2A.Z promotes PRC2 activity by facilitating chromatin compaction, while H3.3 counteracts the positive action of H2A.Z on H3K27me3 deposition by PRC2. Therefore, H3.3 and H2A.Z function coordinately in fine-tuning PRC2 enzymatic activity to control H3K27me3 level through modulating the chromatin structure/compaction. Here we use “Bagua” (also named “the Eight Diagrams”—eight combinations of three whole or broken lines formerly used in divination), which has been used in Taoist cosmology, to iconically summarize the molecular mechanism by which H2A.Z and H3.3 coordinately regulate PRC2 activity and H3K27me3. In our model, each trigram of the four, which originally represents the fundamental principle of reality in Taoist cosmology, represents a range of four interrelated chromatin status as shown in our experiments. In addition, each line either “broken” (highlighted by black bars) or “unbroken” (highlighted by red bars) respectively represents yin or yang, a theory describe how seemingly opposite or contrary forces may actually be complementary, which may perfectly represent the regulatory effects of H2A.Z and H3.3 on chromatin structure and PRC2 activity/H3K27me3 in our study

Both H3.3 and H2A.Z have been shown to modulate H3K27me3 deposition in mES cells [29, 30], but the underlying mechanisms remain unclear. Here, we show that H2A.Z enhances the PRC2 activity through modulating chromatin structure in vitro and in mES cells. In contrast, H3.3 counteracts the positive action of H2A.Z on H3K27me3 deposition by preventing H2A.Z-mediated chromatin folding. Collectively, our in vitro biochemical studies and genomic assays in mES cells support the hypothesis that the coordination between of H2A.Z and H3.3 plays an important role in the transcriptional regulation of developmental genes through modulating chromatin structure and PRC2-dependent H3K27me3 deposition (Fig. 6).

### H2A.Z enhances PRC2 activity on H3K27 methylation through altering chromatin structure

It has been demonstrated that histone variant H2A.Z is essential for embryonic development by impacting chromatin structure and gene expression [9, 44–46]. Recently, H2A.Z and PcG proteins, which were previously considered as widely divergent proteins, have been shown to function together in regulating developmental genes in undifferentiated ES cells and during lineage commitment [16, 20, 29, 31, 32, 47–49]. Consistent with previous results [29, 31], our ChIP-seq analysis shows that in mES cells, H3K27me3 is positively correlated with H2A.Z at genome wide and that H3K27me3 is largely reduced upon knockdown of H2A.Z. The causal relationship between H2A.Z enrichment and PRC2-dependent H3K27me3 deposition is supported by our in vitro enzymatic activity assays showing that H2A.Z directly promotes PRC2 enzymatic activity through facilitating higher-order chromatin folding. Previously, it has been shown that PRC2 complex preferably methylates the oligo-nucleosomal histone H3 at Lysine 27 and PRC2 activity on oligo-nucleosomes is enhanced by the addition of magnesium (Mg^2+^) or H1 [48]. It is well known that both magnesium (Mg^2+^) and H1 can promote the oligo-nucleosomes folding into condensed chromatin fibers. These results suggest that PRC2 enzymatic activity is dependent on the structure of chromatin substrates, that is, condensed chromatin is a preferred substrate for the PRC2 complex. A recent report has confirmed that PRC2 activity indeed is promoted by dense nucleosomal arrays [36]. Interestingly, they also found that PRC2 complex can be further activated by the histone H3 tails (Ala31 to Arg42) of neighboring nucleosomes, which suggests PRC2 activity is dependent on the proximity between its substrate nucleosomes. Of note, we and others have previously demonstrated that H2AZ facilitates the formation of “ladder-like” chromatin fibers in which the neighboring nucleosomes are stacked together, in the presence of MgCl_2_ [16, 22, 37]. Together, our results strongly support a model in which H2A.Z promotes PRC2 activity by promoting the folding of nucleosomal arrays into a dense structure. Consistently, we further demonstrated that H2A.Z acidic patch, which is critical for chromatin folding, is also important for the stimulatory effect on PRC2 activity in vitro and in mES cells. Of note, the acidic patch of H2A.Z has also been shown to play important roles in regulating gene expression programs during mES cells differentiation and *Xenopus laevis* development [45, 50]. Together, our findings provide a rational explanation for how H2A.Z regulates PRC2 complex in mES cells although they do not directly interact [29].

### H2A.Z and H3.3 coordinately regulate PRC2-dependent H3K27me3 deposition in mES cells

Histone variant H3.3 was widely considered as an active chromatin marker [12], but it has also been recently shown to play critical roles in gene silencing and heterochromatin [23, 24]. Previously, we have shown that H3.3 not only impairs the folding of nucleosomal arrays but also counteracts the H2A.Z-enhanced folding of nucleosomal arrays [22]. Therefore, it is tempting to speculate that H3.3 may negatively regulate PRC2-mediated H3K27 methylation through counteracting with H2A.Z. Indeed, our in vitro HMT assays showed that H3.3 impairs the enhanced effect of H2A.Z on PRC2 activity. Our genomic analysis in mES cells shows that loss of H3.3 results in significant increase of H3K27me3 deposition at the major H3K27me3 peak regions (referred to as type I). Moreover, our time course ChIP and DNA nuclease protection analysis showed that H3.3 incorporates into the promoter region of CYP26a1 gene to maintain the local chromatin open at the early phase (phase I) (from 0.5 h to 2 h) after transcriptional cessation. The chromatin accessibility at the promoter region start to reduce when the H3.3 level goes down at the second phase (4 h after transcriptional cessation), then the local compaction of chromatin at the promoter region could promote PRC2-dependent H3K27me3 deposition during gene repression in mES cells. Collectively, our results support a model where H3.3 plays a negative role in regulating PRC2 activity and H3K27me3 deposition by limiting the positive effect of H2A.Z.

Surprisingly, our genomic analysis also showed that loss of H3.3 significantly attenuates H3K27me3 deposition at a subgroup of H3K27me3 peak regions (referred as type II). Interestingly, H2A.Z level was also observed to concordantly decrease at these type II regions, which could well explain the observed effect on H3K27me3 deposition at these type II regions upon loss of H3.3. Of note, our previous study has shown that H3.3 is highly enriched at enhancer regions and can coordinately regulate the distal deposition of H2A.Z into promoter regions [22, 51]. Therefore, we speculate that H3.3 may regulate PRC2 activity and H3K27me3 deposition by controlling the deposition of H2A.Z at the promoters of developmentally regulated genes. Similarly, a recent study showed that the level of H3K27me3 is reduced at bivalent promoters upon H3.3 depletion [30]; however, they argued that PRC2 complex can be recruited to regulate H3K27me3 deposition at the promoters of developmentally regulated genes by HIRA complex in a H3.3-dependent manner. Therefore, it is of great interest to further decipher the molecular mechanism of how H3.3 positively regulates the deposition of H3K27me3 by PRC2 at these type II genomic regions and its possible biological functions in mES cells.

## Conclusions

Together, our results reveal that H3.3 can negatively regulate H3K27me3 deposition through directly counteracting the enhanced effect of H2A.Z on PRC2 activity when H3.3 co-localizes with H2A.Z at the same genomic regions (Type I), on the other hand, H3.3 can also positively regulate H3K27me3 deposition by controlling the distal deposition of H2A.Z at the promoters of developmental genes regulated by PcG complex in mES cells through an unknown mechanism. Therefore, it is of great interest to further decipher the molecular mechanism by which H3.3 at regulatory regions can coordinately control the distal deposition of H2A.Z at the promoters of developmental genes in mES cells.

## Methods

### Protein, DNA, and antibodies

The recombinant histones and DNA templates of 12 tandem 177 bp repeats of 601 sequences and plasmid pG5MPL were cloned and purified as previously described [52]. For in vitro histone methyltransferase activity study, PRC2 complexes were purified as described [53]. The anti-H3K27me3 antibody (07-449) was from Millipore, the anti-H3K27me1 antibody (65015) was purchased from Active Motif, the anti-H3K9me3 antibody (ab8898) was from Abcam, the anti-H3K36me3 antibody (ab9050) was from Abcam, anti-H3 pan antibody (ab1791) was from Abcam, anti-H2A.Z antibody (39113) for Western blot was from Active Motif, anti-H2A.Z antibody (ab4174) for ChIP was from Abcam, and monoclonal Anti-HA Agarose Conjugate Clone HA-7 (A2095) was from Sigma.

### Cell culture

Mouse ES cells (line R1 Mouse Embryonic Stem Cells, SCRC1011) were grown in gelatin-coated tissue culture plates in the presence of 1,000 U/ml of leukemia inhibitory factor (LIF; ESGRO, Millipore) in ES cell medium consisting of knockout Dulbecco’s minimal essential medium (DMEM; GIBCO/BRL) or Dulbecco’s minimal essential medium (DMEM; specialty media) supplemented with 15% FBS (Hyclone), 100 mM MEM nonessential amino acids, 0.55 mM 2-mercaptoethanol, 2 mM l-glutamine, nucleosides, and antibiotics (all from Millipore). Stable overexpressed histone H2A or H2A.Z mouse R1 ES cell line was generated by transfection with pCAG-H2A or H2A.Z plasmids and selected with 1 μg/μl puromycin. All the primary cell lines were tested via MycoAlert™ Mycoplasma Detection Kit (LONZA).

### RNAi-mediated gene silencing and CRISPR-Cas9 mediated gene editing in mES cells

H2A.Z was knocked down by transient transfection with siH2A.Z. In mammals, histone H3.3 is encoded by two different genes (h3f3a and h3f3b) whose transcription results in identical protein products (H3.3A and H3.3B) [54]. H3.3B was knocked out by using CRISPR/Cas9, and H3.3A was knocked down by using siRNA. The double strand siRNA targeting to H2A.Z (forward 5′-GGTAAGGCTGGAAAGGACT-3′; reverse 5′-AGTCCTTTCCAGCCTTACC-3′) and to H3.3A (forward 5′-TGAGTTGTCCTACATACAA-3′; reverse 5′-TTGTATGTAGGACAACTCA-3′) were purchased from Genepharma (Shanghai, China). To generate knockout ES cells, pX260 was modified by inserting the guide sequence site of pX330 [55]. SpCas9 target sites were designed by the CRISPR design software [56]. The targeting sequence of H3.3B is (5′-GTTTGCGGGGGGCTTTCCCACCGG-3′). Plasmids were then transfected into mES cells by Lipofectamine 2000 (Invitrogen) according to the manufacturer’s instructions. Then, the cells were seeded into a 10-cm dish with low confluence, and 12 h later puromycin (InvivoGen) was added to select clones for 10 days. Then individual clones were picked out and screened by PCR followed by Sanger sequencing, and successful knockout was confirmed by RT-qPCR.

### Isolation of mRNA and real-time PCR analysis

Total RNA was extracted using Trizol reagent (Invitrogen, USA), and the first strand of cDNA was reverse transcribed using 2 μg of RNA. cDNA products were used for quantitative real-time PCR using SYBR Premix Ex Taq (Takara, Japan). The sequences of mRNA detecting primers used in RT real-time PCR (ABI 7300, USA) are as follows: H2A.Z (sense 5′-TATCACCCCTCGTCACTTGC-3′, antisense 5′-TCCACTGGAATCACCAA CAC-3′), mH3f3b (sense 5′-CTGAGAGAGATCCGTCGTTACC-3′, antisense 5′-GCTTCAACTTAAGCTCTCTCCC-3′), GAPDH (sense 5′-GCACAGTCAAGGCCGAGAAT-3′, antisense 5′-GCCTTCTCCA TGGTGGTGAA-3′). The results were calculated by three independent experiments.

### MNase-seq analysis

Protocol of MNase-seq is derived from our previous method [22]. GST-tagged MNase protein was expressed and purified from *Escherichia coli* bacterial cells. The genome of mES cells (R1 mES cells, SCRC-1011) was digested with GST-MNase at different time points. Subsequently, the genomic DNA from the GST-MNase digestion was extracted with phenol-chloroform, precipitated with ethanol, and analyzed on a 1.0% agarose gel. A mild digestion condition in which a small fraction of the genome was digested into a mononucleosome is chosen here (Additional file 3: Figure S3A). The DNA fragments corresponding to mononucleosomal sizes of the selected digestion condition were isolated and purified from the gel, and the resulting DNAs were subjected to sequencing using the HiSeq 2500 sequencing system. FASTQ sequences at 36 bp in length were aligned to the *Mus musculus* reference genome (mm9) using BWA with default parameters [57]. Only the uniquely mapped reads with a quality > 10 were considered for genome-wide data analysis. The genome-wide data analyses were performed as described in the “Data analysis” section.

### Chromatin structure analysis in vivo

The chromatin structure was analyzed using the EpiQ™ chromatin analysis kit (Bio-Rad, USA). Briefly, chromatin was digested using DNase I in situ for 1 h. The digested and undigested chromatin DNA was purified and quantified using the Nanodrop 2000 (Thermo, USA). The samples were analyzed using real-time PCR with the following primer sequences: Adra2c (sense 5′-GCAAAGTAAAGTTGCAGGGACC-3′, antisense 5′-CTGGGTTCAGTGGG AGCGTC-3′), Foxf1a (sense 5′-TACATCGCGCTCATCGTCAT-3′, antisense 5′-CGCTAGCC GGATCGATGG-3′), Trim47 (sense 5′-GACACAGGCTCTCCTCAAGTC-3′, antisense 5′-CT CCGGCTCCGGAACCATC-3′), and Rgmb (sense 5′-ATTTACCCCCAAACTCCGGT-3′, antisense 5′-GACACCCCGGGCCTTTATTG-3′). The results were calculated by three independent experiments.

### Chromatin immunoprecipitation of crosslinked mononucleosomes

For a typical experiment, 1–3 × 10^8^ R1 mouse ES cells were harvested and were fixed with 1% formaldehyde at room temperature for 10 min. Cell pellet was washed in PBS, resuspended in cold buffer I (140 mM NaCl, 1 mM EDTA, 50 mM HEPES, pH 7.5, 10% glycerol, 0.5% NP40, 0.25% Triton X-100, 1× protease inhibitors) at a density of 1.5 × 10^7^ cells/ml, incubated at 4 °C for 10 min. The cell suspension was spin down gently at 500 g for 5 min and removes the supernatant. Resuspend the pellet in the same volume previously used of room temperature buffer II (200 mM NaCl, 1 mM EDTA, 0.5 mM EGTA, 10 mM Tris, pH 8.0, 1× protease inhibitors) and incubated at room temperature for 10 min, spin down gently at 500 g for 5 min, and removes the supernatant. Resuspend the pellet in 10 ml buffer III (15 mM NaCl, 60 mM KCl, 0.2 mM CaCl_2_, 10 mM Tris, pH 7.4, 1× protease inhibitors) and incubated at room temperature for 10 min, spin down gently at 500 g for 5 min, and removes the supernatant. The pellet was resuspended in 1 ml buffer III and sonicates with 10 × 30 s pulses at a high setting and was mixed with 100 U micrococcal nuclease (Takara), the chromatin was digested for up to 60 min at 37 °C, and digestion was stopped by the addition of 10 mM EGTA final concentration. Release the mononucleosomes with 3 × 30 s sonication pulses at a low setting. Spin at 14,000*g* for 15 min and move the supernatant to fresh tubes. A fraction of the supernatant did reverse crosslink at 65 °C for more than 8 h and then treated with RNase A and Proteinase K stepwise. DNA was obtained through phenol:chloroform:isopropanol (25:24:1) extraction. The amount of chromatin was calculated by DNA amount, and digestion of chromatin was monitored by separating 500 ng of chromatin DNA on a 1.5% agarose gel (Additional file 1: Figure S1E).

Five hundred micrograms of nucleosomes was diluted 1:5 in incubation buffer (25 mM Tris, pH 8.0, 500 mM KCl, 2 mM EDTA, 10% glycerol, 1× protease inhibitor) and was mixed with 100 μl anti-flag M2 agarose beads (Sigma, 50% slurry, pre-equilibrated with incubation buffer), incubated at 4 °C for more than 16 h. The immunocomplexes were collected by spin down at 500*g* for 10 min and washed five times in the 1 ml wash buffer (as incubation buffer but with 0.1% NP-40). After a final wash, the beads were resuspended in 1× elution buffer (as incubation buffer but with 250 mM flag peptide, Sigma), and the immunocomplexes were eluted at 4 °C for 30 min. The eluted samples were mixed with SDS loading buffer (Tris/glycerol/bromophenol blue), incubated at 95 °C for 10 min, and separated by electrophoresis through an SDS denaturing polyacrylamide gel. Histone modification signals were determined through Western blot.

### Crosslinked ChIP

R1 cells were grown in 150 mm^2^ dishes to 80–90% confluency. Cells were fixed by adding formaldehyde (Sigma) to 1% final concentration; for histone variants and histone modifications, the proper condition is 10 min at room temperature. For H3.3 ChIP by H3F3B-HA tag knockin, mES cells were crosslinked with 1% formaldehyde in DMEM for 10 min at room temperature. Fixation was quenched by addition of 125 mM glycine final concentration. The cells were washed twice with ice-cold PBS and collected by scraping in PBS supplemented with 0.1% NP-40. Cells were pelleted and resuspended in 1 ml buffer I (50 mM Hepes-KOH, pH 7.5, 140 mM NaCl, 1 mM EDTA, 10% glycerol, 0.5% NP-40, 0.25% Triton X-100, 1 mM PMSF, 1× protease inhibitors), incubated at 4 °C for 10 min, and spin down at 1,000 rpm for 5 min. Pellets were collected and resuspended in 1 ml buffer II (200 mM NaCl, 1 mM EDTA, 0.5 mM EGTA, 10 mM Tris, pH 8.0, 1× protease inhibitors), incubated at room temperature for 10 min, and spin down at 1,000 rpm for 5 min. Pellet nuclei were resuspended in 1.3 ml buffer III (1 mM EDTA, 0.5 mM EGTA, 10 mM Tris, pH 8.0, 0.5% *N*-lauroyl-sarcosine, 1× protease inhibitors) and were sonicated with a Bioruptor (Diagnode) at 10 × 30 s sonication pulses at a high setting. The optimized length of chromatin fragments was from 1 to 3 kb. The sonicated samples were centrifuged at 14,000*g* for 15 min and the supernatant containing the soluble chromatin. Aliquot the chromatin fragment suspensions for each immunoprecipitation reaction in a 0.5-ml tube (usually 200 μl per tube), prepare an extra tube with 1/10 of the chromatin to use as input, it will be the 10% input. Add the antibody (2–8 μg), and mix with 100 μl incubation buffer (3% Triton X-100, 0.3% sodium deoxycholate, 6 mM EDTA, 3 mM PMSF, 3× protease inhibitors), incubate at 4 °C for more than 8 h. Add 20 μl of pre-blocked beads (50% slurry) and incubate at 4 °C for 3 h. Wash immunocomplexes six times with 0.4 ml of RIPA buffer (50 mM Hepes, pH 7.6, 10 mM EDTA, 0.7% DOC, 1% NP-40, 0.5 M LiCl, 1 mM PMSF, leupeptin, aprotinin). After a final wash, the beads were resuspended in 300 μl 1× quick wash buffer (10 mM Tris, pH 8.0, 1 mM EDTA, 50 mM NaCl), then elute in 200 μl elution buffer (50 mM Tris, pH 8.0, 10 mM EDTA, 1% SDS) and incubate at 65 °C for 15 min, vortexing every 4 min. Spin to remove all beads and incubate the supernatant at 65 °C for more than 8 h, in order to reverse crosslink. At this point, thaw the input sample, add elution buffer to 200 μl and process as with the IP sample (reverse 1/10 of the amount used for the IP). After reverse crosslink, add 40 μg RNase A to the sample and incubate at 37 °C for 2 h, then add 40 μg Proteinase K and incubate at 55 °C for 30 min. Extract samples with phenol:chloroform:isopropanol (25:24:1, Sigma) and load on a phase lock 1.5 ml Eppendorf tube. DNA precipitates with ethanol buffer (300 mM NaAc, pH 5.2, 2.5 volumes of 100% EtOH, 20 g glycogen (Roche)). Resuspend pellets in 50 μl TE. The samples were analyzed using real-time PCR with the following primer sequences: Cyp26A1 promoter (sense 5′-CGGAACAAACGGTTAAAGATT-3′, antisense 5′-ATAAGGCC GCCCAGGTTA-3′), Cyp26A1 +2000 (sense 5′-TACCCTTGAAGTCTTCCGTG-3′, antisense 5′-GTTGACGATTGTTTTAGTGCC-3′), Adra2c (sense 5′-GGGACCCTAAGCTCCAAAGG-3′, antisense 5′-TGATTGGAGAGTGCGCTTGT-3′), Foxf1a: (sense 5′-CGACACCTACGGCTTC CAG-3′, antisense 5′-CATTCATCATGCCCAAGCCG-3′), Trim47 (sense 5′-CTCGGACAGC GTGTGGTTC-3′, antisense 5′-TCGCTGTCCACTTTGTCAGG-3′), and Rgmb (sense 5′-GGTGGT GGAAACCCGATCC-3′, antisense 5′-AGACACCCCGGGCCTTTAT-3′). The results were calculated by three independent replications.

### ChIP-Seq

ChIP-seq experiments were performed as crosslinked ChIP experiments. DNA libraries were prepared according to the Illumina protocol and sequenced with HiSeq 2500.

### Data analysis

#### Sequence alignment and peak calling

Reads were mapped to the mouse genome (build 37, mm9) using the Burrows-Wheeler Aligner software [57]. Unique reads mapped to a single best matching location with no more than two mismatches were kept for peak identification and profile generation. MACS [58] was used for peak calling in each replicated sample, and the resulting peaks were filtered by peak height to reduce false positives. We computed the total number of reads in individual promoters for comparing histone modifications in different groups of promoters. Read depths were used to normalize peak heights and promoter modification levels across samples. Wiggle files, generated by each 100 bp read, were used to generate average ChIP-seq profiles using the Homer analysis pipeline.

#### Pearson coefficients for the similarity of dynamic change between two epigenetic markers

To quantify the similarity of dynamic changes between H2A.Z and a histone modification H3K27me3, we employed the Pearson coefficient measure [59]. Briefly, the peak summit region was partitioned into 10-bp bins, The tag density of H2A.Z and H3K27me3 was calculated for each bin and parsed into two vectors y and x. Then the Pearson coefficient reads ${r}_{x,y}=\frac{\sum_{i=1}^n\left({x}_i-\overline{x}\right)\left({y}_i-\overline{y}\right)}{\left(\sqrt{\sum_{i=1}^n{\left({x}_i-\overline{x}\right)}^2}\right)\left(\sqrt{\sum_{i=1}^n{\left({y}_i-\overline{y}\right)}^2}\right)}$, when *i* is the index of an element in the vector, *r* can be any value from − 1 to + 1, in which + 1 means a perfect positive linear relationship, − 1 means a perfect negative linear relationship, and 0 means no correlation.

### Histone methyltransferase assay

#### Experimental materials

Mouse Polycomb repressive complex 2 (PRC2) was expressed and purified from the SF9 insect cells by co-infecting the cells with baculoviruses expressing Ezh2, Suz12, Eed, and RbAp46/48. Human GST-tagged Suv39H1 were expressed and purified from *E*. *coli*. Human recombinant histone H2A.Z and other recombinant core histones from Xenopus were expressed in *E*. *coli* and then assembled into histone octamers.

#### Substrates

Recombinant oligo-nucleosomes were assembled via sequential salt dialysis using pG5MPL plasmid DNA and histone octamers.

#### HMT assay

HMT assays were performed according to previous reports [48]. For details, the reaction system including 50 mM of Tris-HCl, pH 8.5, 5 mM of MgCl2, 4 mM of DTT, 1 μg of nucleosome substrates, and 0.5 μM *S*-adenosyl-l-[methyl-3H] methionine (3H-SAM) was used for each reaction, and enzyme of PRC2 was titrated from 50 to 200 ng. The reactions were incubated for 60 min at 30 °C.

### Sedimentation velocity analytical ultracentrifugation

For in vitro structural investigation, chromatin arrays were assembled by salt-dialysis method as described previously [60]. The reconstitution reaction mixture with octamers and 601 based DNA templates in TEN buffers (10 mM Tris-HCl, pH 8.0, 1 mM EDTA, 2 M NaCl) were dialyzed over 16 h at 4 °C in TEN buffer, which is continuously diluted by slowly pumping in TE buffer (10 mM Tris-HCl, pH 8.0, 1 mM EDTA) to lower the concentration of NaCl from 2 to 0.6 M.

Chromatin samples were prepared in the measurement buffer (10 mM HEPES, pH 8.0, 0.1 mM EDTA) with 1.2 mM of MgCl_2_. Sedimentation experiments were performed on a Beckman Coulter ProteomeLab XL-I using a four-hole An-60Ti rotor. The samples with the initial absorbance at 260 nm of about 0.5–0.8 were equilibrated for 2 h at 20 °C under vacuum in the centrifuge before sedimentation. Absorbance at 260 nm was measured in continuous scan mode during sedimentation at 20,000 rpm in 12 mm double-sector cells. The data were analyzed by enhanced van Holde-Weischet analysis using Ultrascan II 9.9 revision 1504. S_20,w_ values (sedimentation coefficient corrected for water at 20 °C) were calculated with a partial specific volume of 0.622 ml/g for chromatin and buffer density and viscosity adjusted. The average sedimentation coefficients were determined at the boundary midpoint.

### Data Availability

The raw files from both ChIP-Seq and MNase Hypersensitive Sites-Seq (MHS-Seq) have been deposited in the NCBI Sequence Read Archive (SRA) database or NCBI Gene Expression Omnibus (GEO) database. They are accessible through SRA accession number SRP154023 or GEO accession number GSE117035.

## Additional files

Additional file 1: **Figure S1.** H2A.Z is required for the proper genome-wide distribution of H3K27me3 in mES cells. (PDF 2983 kb)

Additional file 2: **Figure S2.** H2A.Z is required for the proper genome-wide distribution of H3K27me3 in CD4^+^ T cells. (PDF 2416 kb)

Additional file 3: **Figure S3.** Promotion of PRC2 enzymatic activity by H2A.Z through facilitating chromatin compaction. (PDF 1206 kb)

Additional file 4: **Figure S4.** Dynamic changes of chromatin compaction and H3K27me3 deposition upon knockdown of H2A.Z in mES cells. (PDF 1419 kb)

Additional file 5: **Figure S5.** The extended acid patch of H2A.Z is important for the proper H3K27me3 level in mES cells. (PDF 1852 kb)

Additional file 6: **Figure S6.** Coordination between H3.3 and H2A.Z in regulating H3K27me3 deposition in mES cells. (PDF 1029 kb)
