# Supplementary material for: Histone variants H2A.Z and H3.3 coordinately regulate PRC2-dependent H3K27me3 deposition and gene expression regulation in mES cells
Source: BMC Biol. 2018 Sep 24;16:107. doi: 10.1186/s12915-018-0568-6 (PMC6151936; doi:10.1186/s12915-018-0568-6)
Supplement: Supplementary file 1 — Figure S1. H2A.Z is required for the proper genome-wide distribution of H3K27me3 in mES cells. (PDF 2983 kb) [file 12915_2018_568_MOESM1_ESM.pdf]

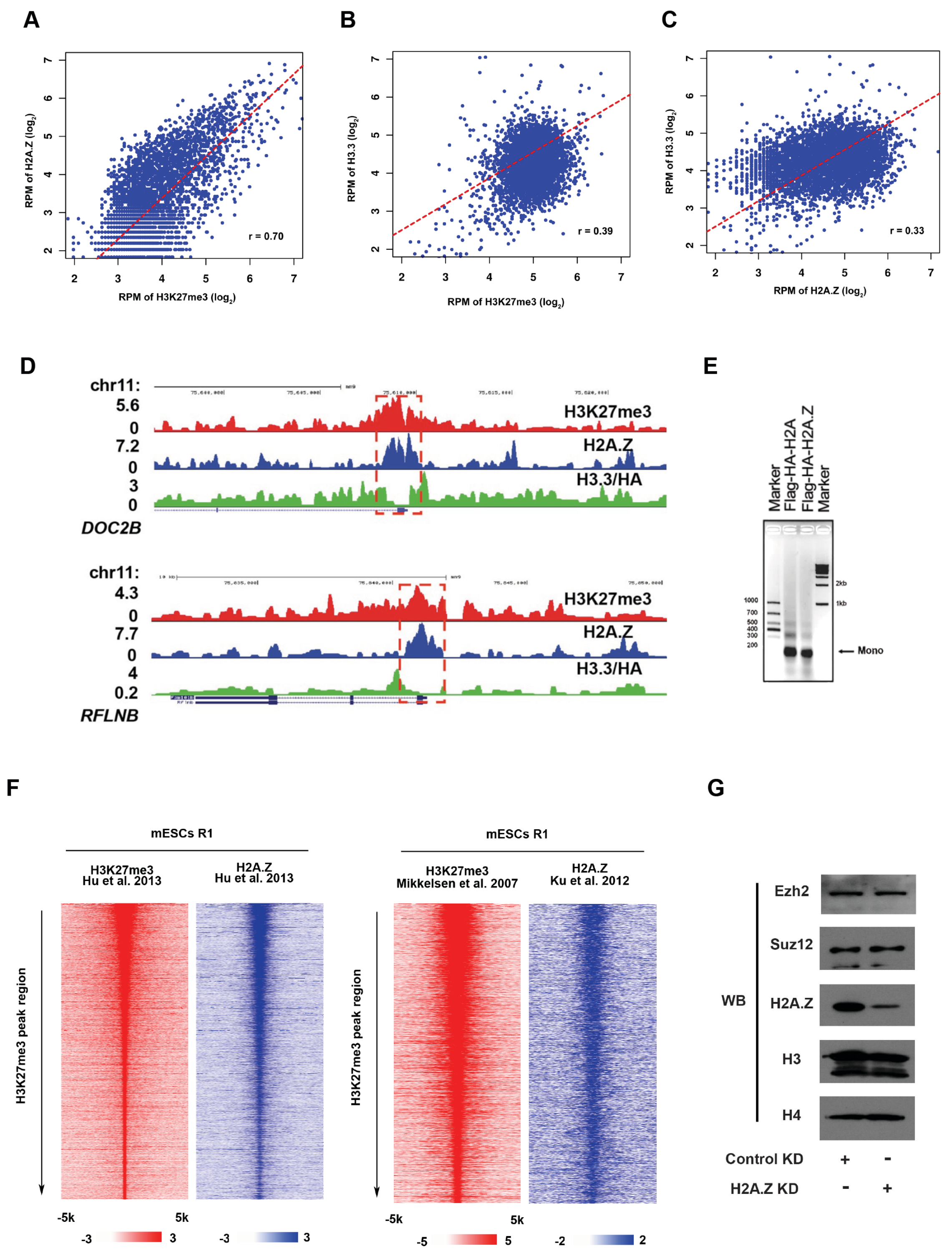

Additional file1: Fig.S1. H2A.Z is required for the proper genome-wide distribution of H3K27me3 in mES cells.

A. Scatterplot comparing the mean of the H3K27me3 and H2A.Z ChIP–seq signals on H3K27me3 peak regions. Both signals of H3K27me3 and H2A.Z are normalized with RPM. The coefficient of determination (r) is indicated.

B. Scatterplot comparing the mean of the H3K27me3 and H3.3 ChIP–seq signals on H3K27me3 peak regions. Both signals of H3K27me3 and H2A.Z are normalized with RPM. The coefficient of determination (r) is indicated.

C. Scatterplot comparing the mean of the H2A.Z and H3.3 ChIP–seq signals on H3K27me3 peak regions. Both signals of H3K27me3 and H2A.Z are normalized with RPM. The coefficient of determination (r) is indicated.

D. H3K27me3, H2A.Z and H3.3 occupancy in specific gene loci (from top to bottom: *DOC2B, RFLNB respectively*) in wild-type mES cells.

E. DNA electrophoresis used to check the quality of H2A- or H2A.Z-containing mono-nucleosomes in mono-nucleosomes immune-precipitation (Mono-IP) assay.

F. Heat-map for distributions of H3K27me3, H2A.Z at the H3K27me3 enriched regions in mES cells. ChIP-seq data was cited from Ku M. et al., 2012 and Hu G. et al., 2013 correspondingly.

G. Western blot to analyze the dynamic changes of Ezh2 or Suz12 protein level upon knockdown of H2A.Z in mES cells.
