# Supplementary material for: Histone variants H2A.Z and H3.3 coordinately regulate PRC2-dependent H3K27me3 deposition and gene expression regulation in mES cells
Source: BMC Biol. 2018 Sep 24;16:107. doi: 10.1186/s12915-018-0568-6 (PMC6151936; doi:10.1186/s12915-018-0568-6)
Supplement: Supplementary file 2 — Figure S2. H2A.Z is required for the proper genome-wide distribution of H3K27me3 in CD4+ T cells. (PDF 2416 kb) [file 12915_2018_568_MOESM2_ESM.pdf]

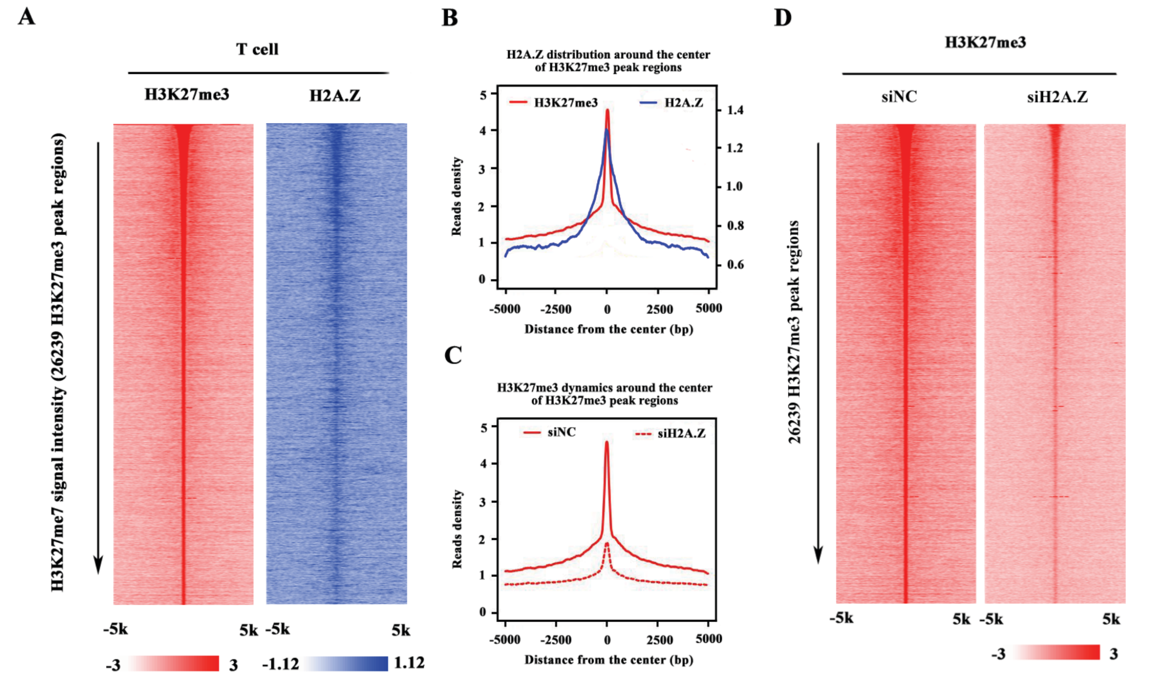


**Additional file2: Fig. S2. H2A.Z is required for the proper genome-wide distribution of H3K27me3 in CD4$^{+}$ T cells.**

**A.** Heat-map for the distribution of H3K27me3 and H2A.Z around H3K27me3 total peak regions in T lymphocyte cells. The Y-axis coordinates with 26,239 high confident H3K27me3 peaks. The zero point of X-axis corresponds with center of each H3K27me3 peak and expands from 5kb upstream to 5kb downstream to form H3K27me3 peak regions.

**B.** Scatterplot exhibits the average reads density of H3K27me3 (red line) and H2A.Z (blue line) around the H3K27me3 peak regions.

**C.** Scatterplots for average reads densities of H3K27me3 around H3K27me3 peak regions in wild-type T cells (referred as siNC) and H2A.Z knocking down T cells (referred as siH2A.Z).

**D.** Heat-map for dynamic changes of H3K27me3 levels around H2A.Z/H3K27me3 co-peak regions upon knockdown of H2A.Z in T cells. The zero point of X-axis corresponds with center of each H3K27me3 peak and expands from 5kb upstream to 5kb downstream to form H3K27me3 peak regions.
