# Supplementary material for: Histone variants H2A.Z and H3.3 coordinately regulate PRC2-dependent H3K27me3 deposition and gene expression regulation in mES cells
Source: BMC Biol. 2018 Sep 24;16:107. doi: 10.1186/s12915-018-0568-6 (PMC6151936; doi:10.1186/s12915-018-0568-6)
Supplement: Supplementary file 3 — Figure S3. Promotion of PRC2 enzymatic activity by H2A.Z through facilitating chromatin compaction. (PDF 1206 kb) [file 12915_2018_568_MOESM3_ESM.pdf]

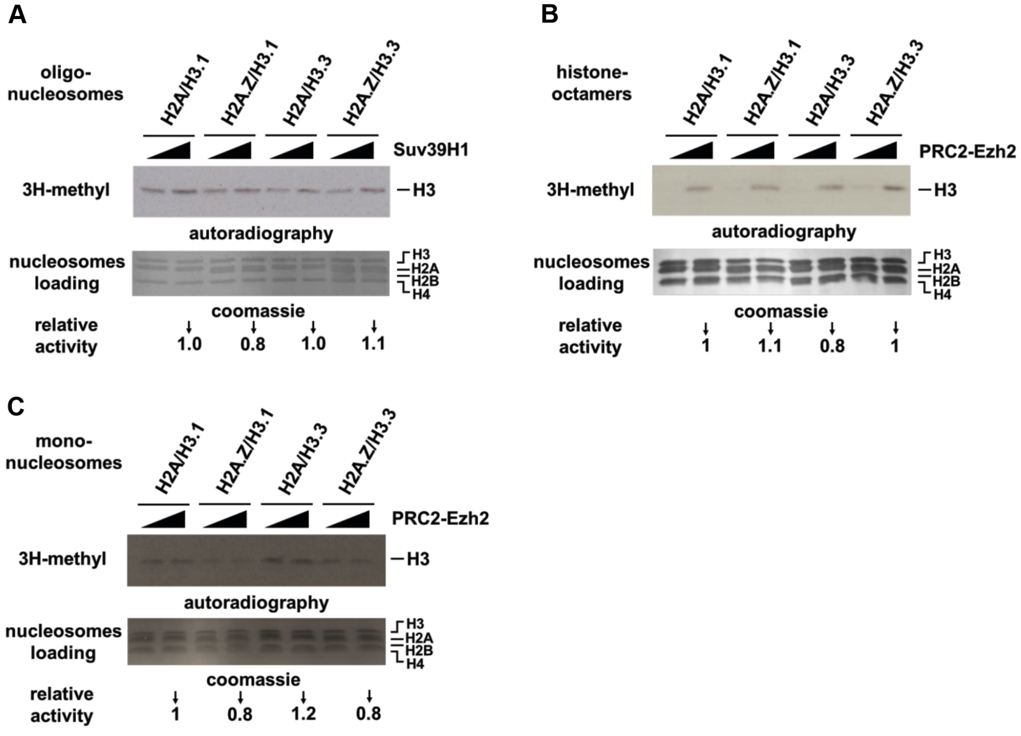

Additional file3: Fig. S3. Promotion of PRC2 enzymatic activity by H2A.Z through facilitating chromatin compaction.

A. Suv39H1 histone methyltransferase activity *in vitro* on the oligo-nucleosomal substrates containing different combination of histone variants (H2A/H3.1, H2A.Z/H3.1, H2A/H3.3, and H2A.Z/H3.3).

B. PRC2 histone methyltransferase activity *in vitro* on the histone-octamers substrates containing different combination of histone variants (H2A/H3.1, H2A.Z/H3.1, H2A/H3.3, and H2A.Z/H3.3).

C. PRC2 histone methyltransferase activity in vitro on the mononucleosomal substrates containing different combination of histone variants (H2A/H3.1, H2A.Z/H3.1, H2A/H3.3, and H2A.Z/H3.3).
