# Supplementary material for: Histone variants H2A.Z and H3.3 coordinately regulate PRC2-dependent H3K27me3 deposition and gene expression regulation in mES cells
Source: BMC Biol. 2018 Sep 24;16:107. doi: 10.1186/s12915-018-0568-6 (PMC6151936; doi:10.1186/s12915-018-0568-6)
Supplement: Supplementary file 4 — Figure S4. Dynamic changes of chromatin compaction and H3K27me3 deposition upon knockdown of H2A.Z in mES cells. (PDF 1419 kb) [file 12915_2018_568_MOESM4_ESM.pdf]

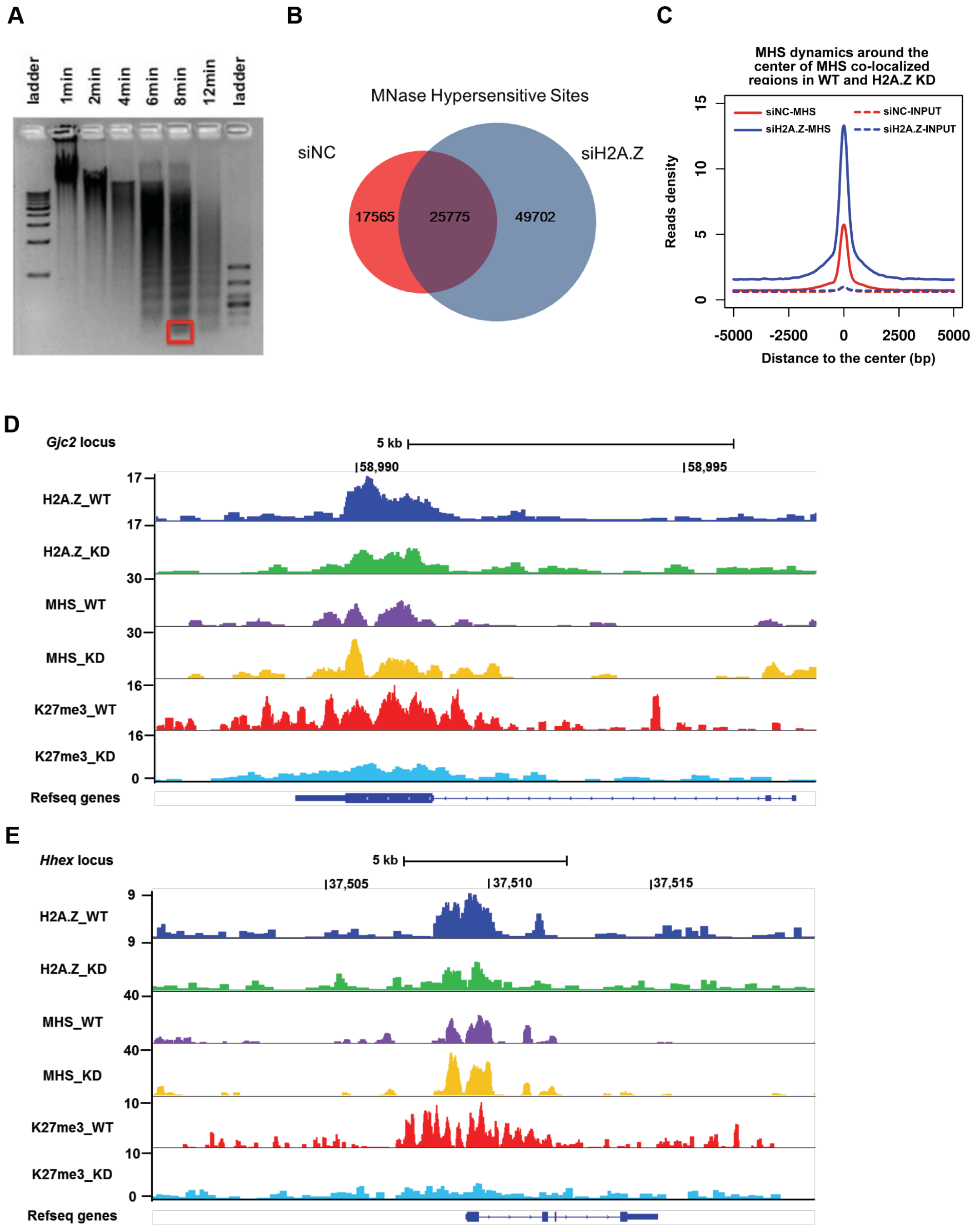

Additional file4: Fig. S4. Dynamic changes of chromatin compaction and H3K27me3 deposition upon knockdown of H2A.Z in mES cells.

A. Agarose gels demonstrate GST-MNase digestion ladders. To obtain mono-nucleosomes from partial digestion, DNA fragments (size between 100bp and 200bp) from the lane of “8min” (highlighted by red frame) were chosen for deep sequencing.

B. Venn-diagrams show overlaps of MNase hypersensitive peak sites (MHS) in both wild type mES cells (referred as siNC) and H2A.Z knocking down mES cells (referred as siH2A.Z). Data from MNase extensive digestion assay were used as “Input” in peak calling process.

C. Scatterplot exhibits average reads densities of MNase hypersensitive levels (MHS) around MHS overlapping sites (marked by Figure S3A) in wild type mES cells (referred as siNC-MHS) and H2A.Z knocking down mES cells (referred as siH2A.Z-MHS). The nucleosome occupancy profile for each cells were showed as well (referred as siNC-INPUT and siH2A.Z-INPUT).

D-E. The dynamic changes of levels of H2A.Z, MHS and H3K27me3 in specific gene loci (Gjc2 and Hhex) upon knockdown of H2A.Z in mES cells.
