# Supplementary material for: Histone variants H2A.Z and H3.3 coordinately regulate PRC2-dependent H3K27me3 deposition and gene expression regulation in mES cells
Source: BMC Biol. 2018 Sep 24;16:107. doi: 10.1186/s12915-018-0568-6 (PMC6151936; doi:10.1186/s12915-018-0568-6)
Supplement: Supplementary file 5 — Figure S5. The extended acid patch of H2A.Z is important for the proper H3K27me3 level in mES cells. (PDF 1852 kb) [file 12915_2018_568_MOESM5_ESM.pdf]

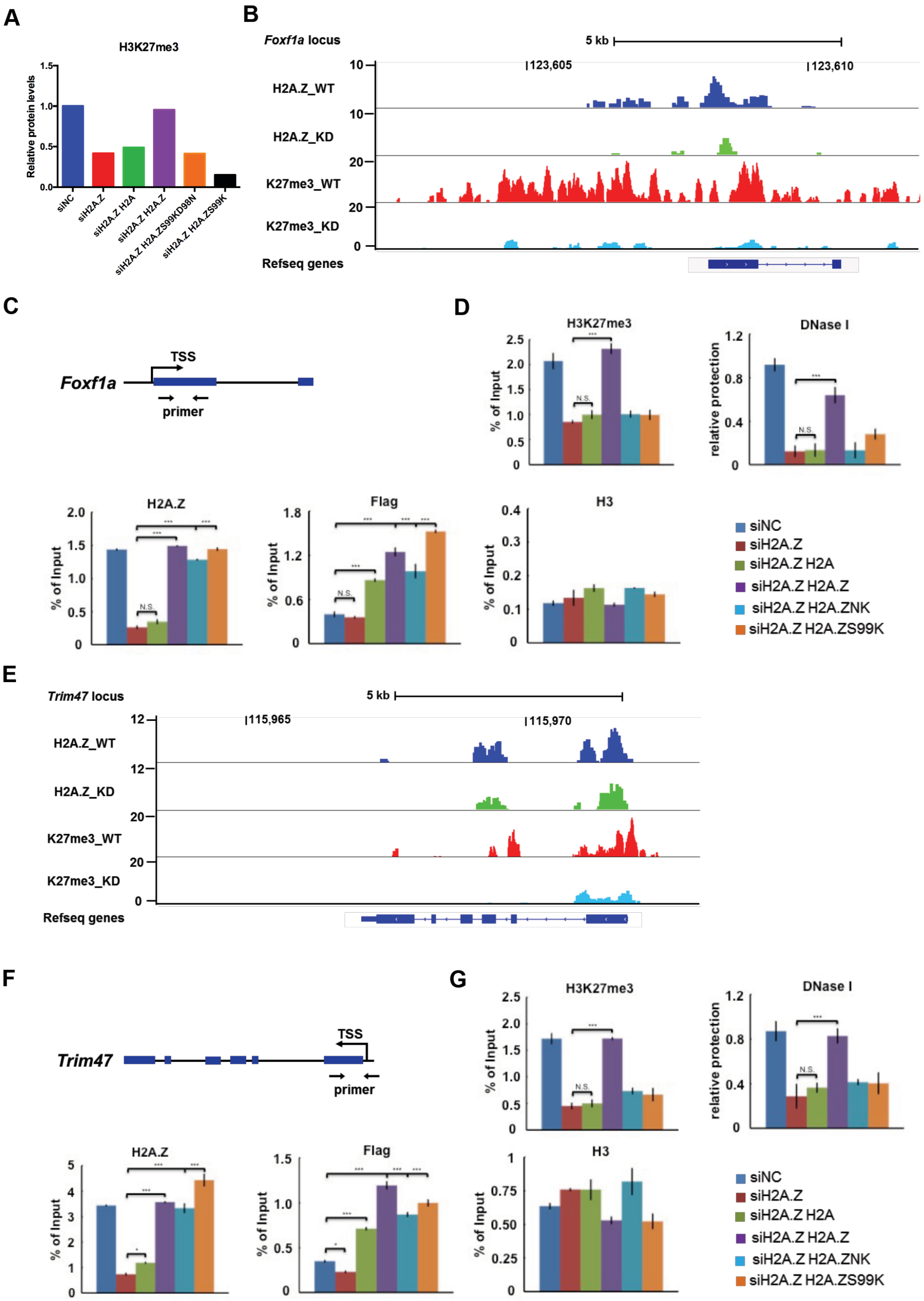

Additional file5: Fig. S5. The extended acid patch of H2A.Z is important for the proper H3K27me3 level in mES cells.

A. Quantitative analysis of western-blot coordinate in figure 4A.

B. The dynamic changes of levels of H3K27me3 and H2A.Z in specific gene loci (*Foxf1a*) upon knockdown of H2A.Z in mES cells.

C. Schematic view of *Foxf1a* promoter region, primer pairs are indicated by arrows.

D. Rescuing the deposition of H3K27me3 and the local chromatin compaction at the promoter of *Foxf1a* in H2A.Z knocking down mES cells by indicated histone or its mutants (H2A, H2A.Z, H2A.Z D98N/S99K and H2A.Z S99K). Levels of H3K27me3, H2A.Z, exogeneous Flag-H2A.Z and H3 at the promoter of *foxf1a* were monitored by ChIP-pPCR. The local chromatin compaction/dynamics at the promoter of *Foxf1a* was analyzed by DNAse protection assay via Epi Q^{TM} kit. The P values were calculated with Student's t-test (***<0.01; *<0.05; n=3).

E. The dynamic changes of levels of H3K27me3 and H2A.Z in specific gene loci (*Trim47*) upon knockdown of H2A.Z in mES cells.

F. Schematic view of *Trim47* promoter region, primer pairs are indicated by arrows.

G. Rescuing the deposition of H3K27me3 and the local chromatin compaction at the promoter of Trim47 in H2A.Z knocking down mES cells by indicated histone or its mutants (H2A, H2A.Z, H2A.Z D98N/S99K and H2A.Z S99K). Levels of H3K27me3, H2A.Z, exogeneous Flag-H2A.Z and H3 at the promoter of foxf1a were monitored by ChIP-pPCR. The local chromatin compaction/dynamics at the promoter of Trim47 was analyzed by DNase protection assay via Epi QTM kit. The P values were calculated with Student's t-test (***<0.01; *<0.05; n=3).
