# Supplementary material for: Histone variants H2A.Z and H3.3 coordinately regulate PRC2-dependent H3K27me3 deposition and gene expression regulation in mES cells
Source: BMC Biol. 2018 Sep 24;16:107. doi: 10.1186/s12915-018-0568-6 (PMC6151936; doi:10.1186/s12915-018-0568-6)
Supplement: Supplementary file 6 — Figure S6. Coordination between H3.3 and H2A.Z in regulating H3K27me3 deposition in mES cells. (PDF 1029 kb) [file 12915_2018_568_MOESM6_ESM.pdf]

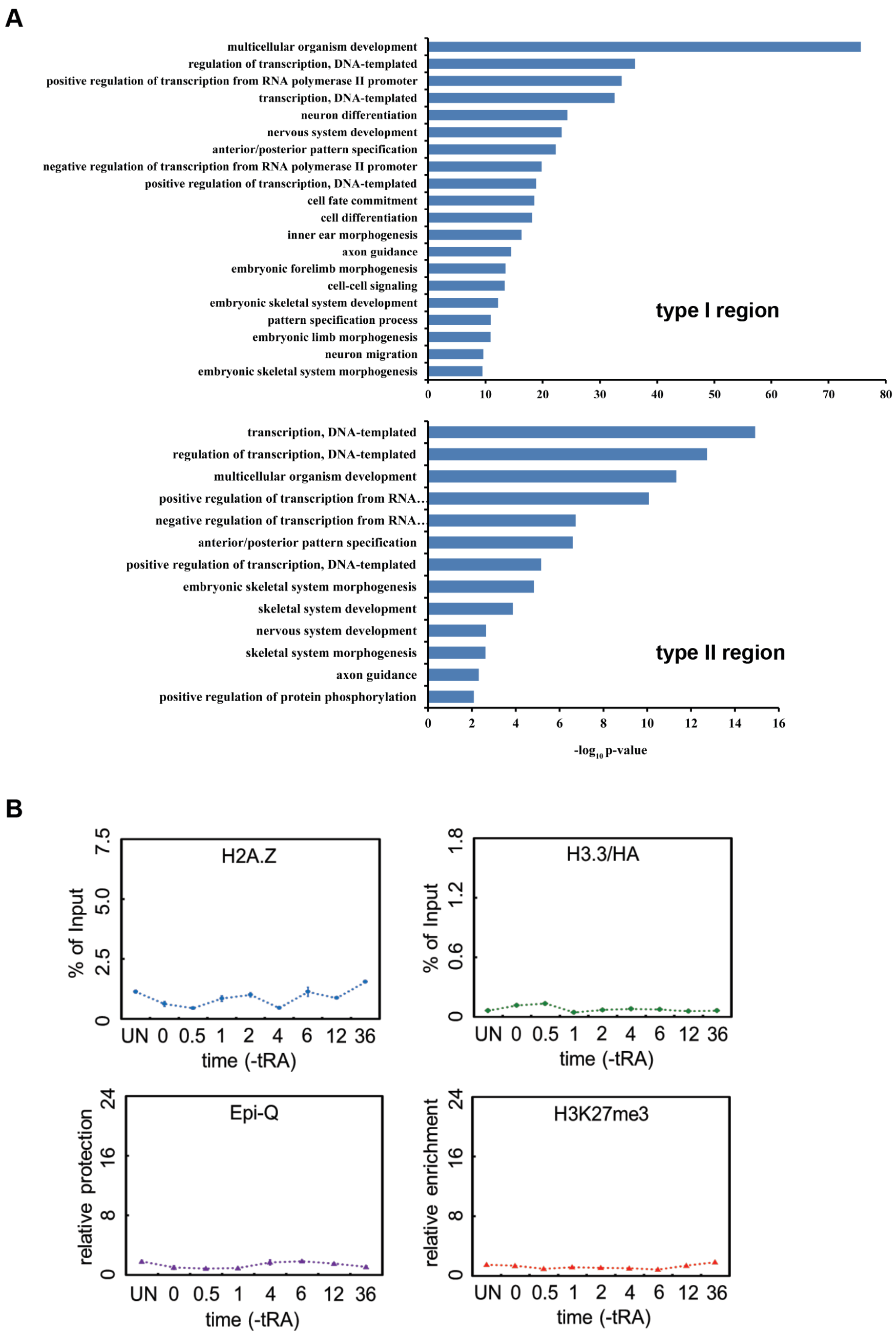

Additional file6: Fig. S6. Coordination between H3.3 and H2A.Z in regulating H3K27me3 deposition in mES cells.

A. Functional annotation of type I and type II H3K27me3 peak regions (as shown in Figure 5B) by BinGO56, which indicates enrichment of genes involved in developmental processes. The x axis values correspond to p-value.

B. The dynamics of H2A.Z (upper left), H3.3 (upper right, represented by HA), chromatin structure/compaction (bottom left) and H3K27me3 (bottom right) at the genebody regions of CYP26a1 during tRA cessation.
